# Control-guided refinement of partially specified Boolean networks: applications to RTK signaling

**DOI:** 10.1093/bioinformatics/btag275

**Published:** 2026-05-24

**Authors:** Eva Šmijáková, Luboš Brim, Samuel Pastva, David Šafránek

**Affiliations:** Faculty of Informatics, Masaryk University, Brno 60200, Czech Republic; Faculty of Informatics, Masaryk University, Brno 60200, Czech Republic; Faculty of Informatics, Masaryk University, Brno 60200, Czech Republic; Faculty of Informatics, Masaryk University, Brno 60200, Czech Republic

## Abstract

**Motivation:**

System control can be used to provide new insights into the dynamics of biological systems. A key application is the identification of therapeutic targets *in silico*, which requires an executable model of the system’s dynamics. However, such models are typically underspecified due to incomplete mechanistic knowledge.

**Results:**

We introduce a novel computational framework that employs control-guided model refinement, predicting informative perturbation experiments to reduce knowledge gaps. The approach is based on partially specified Boolean networks (PSBNs), which enable direct integration of uncertain or incomplete information into executable models. We further extend the framework to handle oscillatory phenotypes as explicit control targets. The applicability of the method is demonstrated on receptor-tyrosine kinase (RTK) signaling, with a focus on fibroblast growth factor signaling in the context of skeletal dysplasias and cancer. We obtain several new insights into modelling of the FGFR3–MAPK pathway.

**Availability and implementation:**

Code and datasets are available at https://doi.org/10.5281/zenodo.16886813.

## 1 Introduction

Boolean networks (BNs) are an abstract modelling framework used in systems biology to describe qualitative dynamics of gene regulation and signaling (Rozum *et al*. 2024). BNs simplify molecular components such as receptors, kinases, and transcription factors into binary variables (active or inactive). Logical rules (*update functions*) determine their interactions.

Unfortunately, available experimental data and prior knowledge are not always sufficient to fully identify the BN of a chosen system. A challenge in BN modelling is therefore model *discrimination and refinement*. That is, determining which network structure or logic is accurate, and devising perturbation experiments that rule out spurious models. An efficient strategy for experiment design is needed to optimally select such perturbations (Videla *et al*. 2015).

To address the challenge of modelling with incomplete knowledge, we rely on *partially specified* Boolean networks (PSBN) (Beneš *et al*. 2023a). PSBNs encode ensembles of candidate models with identical BN structure while adhering to shared but incomplete update rules. PSBNs are thus suitable for model refinement, as the incomplete rules can be gradually specialized through iterative experiments.

BN models enable the prediction and analysis of *attractors*—sets of states to which the model converges over time. Attractors are commonly associated with biological phenotypes (Rozum *et al*. 2024) and often serve as control targets—states to which the system can be driven by interventions (Zañudo and Albert 2015, Murrugarra *et al*. 2016, Moradi *et al*. 2019). However, permanent *perturbation* (such as gene over-expression or knock-out) can disrupt the original long-term behavior, possibly causing emergence of new attractors (Su and Pang 2021).

To account for this option, *phenotype control* proposes to use a set of model traits as the control target. Instead of requiring a specific attractor repertoire, only the phenotypic traits of attractors are assessed. Here, we base our description of phenotype control on Choo *et al*. (2018) and Fontanals *et al*. (2020). Existing work on phenotype control (e.g. Paulevé 2023) focuses on *stabilization* in a given phenotype (i.e. all attractor states must adhere to the chosen traits). However, in many cases, observed phenotypic traits describe stages within a larger *oscillation*, e.g. circadian clock, cardiac rhythms, or cell cycles (Vecchio *et al*. 2016, Li and Yang 2018). Moreover, qualitative abstraction in BNs can cause certain steady states to appear as oscillations in the Boolean domain. Morris *et al*. (2010) demonstrate this behavior in signaling pathways, further motivating oscillatory phenotypes as explicit control targets.

**Figure 1:**
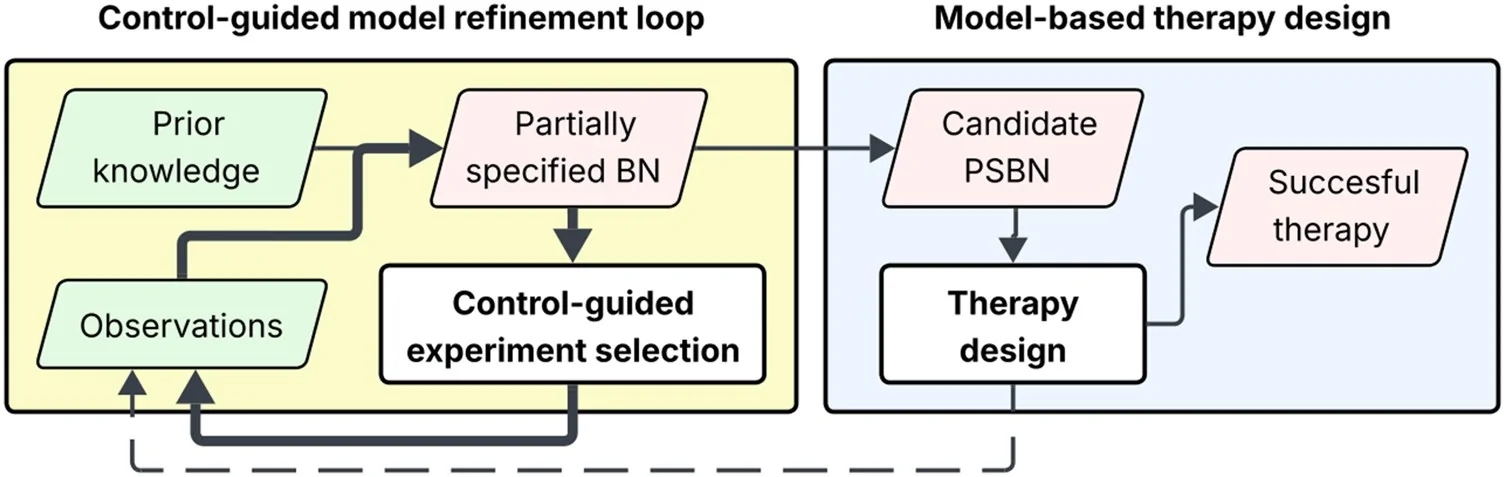
Applications of BN control. Traditional use of control is in therapy design (right), driving the system toward a desired phenotype. This paper focuses on control-guided model refinement (left; specifically the loop consisting of thick arrows), improving candidate model quality and by extension, the likelihood of later successful therapy design.

Traditional use of control is in therapy design, robustly driving the system toward the desired phenotype. While therapy design can generate new experiments that inform the partially specified model, we focus on explicit usage of phenotype control in the model refinement loop (Fig. 1). Our contributions are the following: First, we expand existing symbolic control approach for PSBNs (Beneš *et al*. 2023b) to allow traits arising in oscillatory phenotypes (relevant for both model refinement and therapy design). Then, we propose a workflow which applies this new method to model refinement, deriving perturbation experiments that optimally reduce the set of candidate models, and specific hypotheses about PSBN features that can be confirmed or refuted using these experiments. To show the applicability of our approach, we present a case study that targets signaling pathways related to cell growth, confirming recent experimental observations.

### 1.1 MAPK cell growth signaling

We focus on pathways of receptor tyrosine kinases which bind growth factor ligands, playing key role in cellular growth, differentiation, metabolism, and motility (Hubbard and Miller 2007). We specifically address activation mechanisms targeting mitogen-activated protein kinases (MAPK) that form signaling cascades driving cell cycle control, proliferation, and apoptosis.

Several MAPK signaling pathways have been extensively explored in literature (Cho *et al*. 2016, Traynard *et al*. 2016, Eduati *et al*. 2020). Their pivotal component is Extracellular Signal-Regulated Kinase (ERK), acting as key mediator in signal transmission from activated cell surface receptors to the nucleus (Plotnikov *et al*. 2011). Functions of ERK involve phosphorylating various substrates, including important transcription factors and other proteins, most of them driving *cell proliferation* (Mebratu and Tesfaigzi 2009). Grieco *et al*. (2013) developed a comprehensive logical MAPK signaling model to simulate cell-cycle-related decisions such as proliferation, growth arrest, or apoptosis. These decisions are determined by various growth factor stimuli in conjunction with the cell’s ability to detect DNA damage.

We investigate one particular stimulus—fibroblast growth factor (FGF)—and its downstream signaling mechanisms affecting growth-related phenotypes. In particular, we focus on signaling caused by fibroblast growth factor receptor 3 (FGFR3), known for its critical role in progression of serious developmental diseases (Foldynova-Trantirkova *et al*. 2012, Fafilek *et al*. 2022). First, based on recent biological findings (see below), we build and refine a novel PSBN model of the FGFR3-MAPK signaling isolated from other cellular processes. Subsequently, we extend the existing Grieco *et al*. (2013) BN with our refined FGFR3-MAPK signaling pathway. Control-guided refinement is subsequently used to address the underspecified parts of the model resulting from this extension.

Our FGFR3-MAPK model incorporates recently observed disruption in the negative feedback loop of ERK-FRS2 reported in multiple cancer cell types (Wu *et al*. 2003, Lake *et al*. 2016, Zakrzewska *et al*. 2019). Furthermore, we include the signaling component SHIP2, absent in previous models. Its regulatory role is recognized in chondrocytes, cervical cells, and other cancer-prone tissues (Fafilek *et al*. 2018, Azzi 2020).

The refinement of the isolated FGFR3-MAPK model eliminates a significant portion of model candidates (from hundreds to tens), and reaffirms the suppression of ERK activity caused by SHIP2 knock-out (Fafilek *et al*. 2018). In the extended model, the main insight is a confirmed correlation between long-term ERK dynamics and proliferation/apoptosis. Moreover, we computationally confirm the robust effect of PLCG perturbations on proliferation (Vaqué *et al*. 2014).

### 1.2 Related work

The idea of model refinement informed by control results has been explored before: Ud-Dean and Gunawan (2016) design knockout and over-expression experiments to refine gene regulatory networks. Similar strategies exist for Boolean networks, but they primarily focus on modelling input–output behavior, lacking the generality of our method. Videla *et al*. (2015) deal with synchronous BNs without feedback loops. Gjerga *et al*. (2020) combine discrete and continuous semantics achieving promising results in refinement of signaling pathway models, but assess long-term dynamics only via simulation. Similarly, Atias *et al*. (2014) target selection of experiments with maximum entropy based on BN simulation. However, the use of simulation limits the scalability and completeness of these methods, especially in the context of large asynchronous BNs.

Individual perturbation experiments are often used as inputs for model inference. However, such methods cannot guide selection of new perturbation experiments. Notable examples include Yordanov *et al*. (2016), who employ SMT (Satisfiability Modulo Theories) to constrain systems dynamics, including known perturbation effects. Similarly, Paulevé (2023) uses Answer Set Programming (ASP) to handle constraint-based inference of partially specified models, including the possibility to encode (and synthesize) effects of perturbations. Moreover, this approach addresses the most permissive semantics, which is a coarser over-approximation of the asynchronous update used in this paper. To the best of our knowledge, both methods also struggle to reliably express oscillatory phenotypes.

The underlying symbolic method expands on our previous work in PSBN-based therapy design rooted in phenotype control (Beneš *et al*. 2023b). Here, the method is enhanced with support for oscillatory phenotype traits, used in the fully novel control-guided model refinement framework. The main utility of our framework is its ability to obtain quantitative insights into the solution space of the perturbation problem: While automated reasoning (such as Yordanov *et al*. 2016 and Paulevé 2023) relies on solution enumeration (intractable for large solution spaces), our method produces a symbolic encoding of the complete solution space. It allows us to compute features such as robustness or information gain for candidate perturbations, which then guide the model refinement.

## 2 Preliminaries

First, let us briefly introduce the concepts concerning partially specified Boolean networks and control (formal definitions and examples appear in Section 1, available as supplementary data at *Bioinformatics* online).

### 2.1 Notation

We write B and $B_{⋆}$ to denote the domains {0,1} and {0,1,⋆}. Here, ⋆ is a *free* value (i.e. neither 1 nor 0). The sets of *n*-element vectors $B^{n}$ and $B_{⋆}^{n}$ represent Boolean *states* and *subspaces*. Considering *n* indexed Boolean variables, a state $x\in B^{n}$ assigns a Boolean value to each variable (denoted $x_{i}$ for the *i*-th variable). Meanwhile, a subspace $X\in B_{⋆}^{n}$ represents a state hypercube $S(X)=\{x\in B^{n}|X_{i}\in B\Rightarrow x_{i}=X_{i}\}$.

### 2.2 Boolean networks

A *Boolean network* (BN) is a collection of update functions, one for each variable: $F=\{f_{1},\ldots ,f_{n}\}$ where $f_{i}:B^{n}\to B$. To define BN dynamics, we consider the *asynchronous* update: given a state $x\in B^{n}$, a Boolean model non-deterministically chooses *i* s.t. fi(x)=xi, and transitions into a new state $x^{\prime }=x[i\leftarrow f_{i}(x)]$ (denotes a substitution of the *i*-th value in *x* for $f_{i}(x)$). A directed graph having network states as vertices and the aforementioned non-deterministic transitions as edges is called the *state-transition graph*, denoted STG(F). When interpreting a BN, we focus on sets of states that cannot be escaped within STG(F) (called *trap sets*). Inclusion-minimal trap sets (called *attractors*) are of particular interest.

### 2.3 Network phenotypes

A BN can exhibit many attractors with seemingly identical biological functionality, motivating the concept of *network phenotypes*. Informally, a phenotype is a set of observable characteristics of an organism, resulting from the interaction of its genetic makeup (genotype) with the environment (Johannsen 1911). In the context of BNs, the characteristics are represented by a subset of variables, which we call the *character*. The manifestation of the character, called *trait*, is an assignment of Boolean values to these variables. A combination of traits is then called a *phenotype* Φ. Note that traits correspond to subspaces, meaning a phenotype is a combination of subspaces (see also overview in Table 1). Finally, attractors dictate the relationship between F and Φ: Attractor *A stabilizes in* Φ (resp. *avoids* Φ) when A∩Φ=A (resp. A∩Φ=∅). *A* can also *oscillate through* Φ if it neither avoids nor stabilizes in Φ (implying ∅=(A∩Φ)=A). If *all* attractors follow a particular relationship, the relationship holds for the whole BN.

**Table 1:** Phenotype related terms.^a^

Concept	Real-world	Example	Network	Example
Character	Properties of interest	Presence of FRS2 tyrosine residue phosphorylation	Set of BN variables	{FRS2}
Trait	Observation of properties	FRS2 tyrosine residue is not phosphorylated	Character valuation; alternatively a subspace	{FRS2 ↦ 0} ⋆0⋆
Phenotype	Observation combination	FRS2 or ERK is phosphorylated	Combination of traits	⋆ 1 ⋆ ∪ ⋆ ⋆ 1

a The table explaining the notions of character, trait, and phenotype in the context of real-world concepts in Boolean network models. The considered example is a small excerpt from the FGFR3-MAPK pathway in our case study.

### 2.4 Phenotype control

A *variable perturbation* is determined by a subspace $Q\in B_{⋆}^{n}$. Variables where $Q_{i}=⋆$ are *unperturbed*. Other variables are *perturbed* to a constant $Q_{i}$. The size of *Q* is the number of perturbed variables (zero-sized perturbation is written as Ø). Under perturbation, the BN dynamics are constrained to the subspace *Q*, removing any outgoing transitions, thus ensuring the perturbed variables remain set to their constant values. We denote such *perturbed state-transition graph* STG[Q](F). We say that *Q controls* F toward {stabilisation,oscillation,avoidance} in Φ if all attractors of STG[Q](F) have this relationship with Φ. However, applying a perturbation can shrink existing attractors (when *A* only partially overlaps *Q*), or even create new ones (when *A* can only be escaped by updating the perturbed variables). Mapping out the relationships between perturbations and phenotypes is therefore a complex problem. Finally, for a perturbation to be useful, it must be realizable in practice. The problem of phenotype control therefore implicitly assumes some initial set of candidate perturbations $Q\subseteq B_{⋆}^{n}$ that are biologically feasible. This Q is typically obtained by selecting perturbable variables or limiting the perturbation size (Su and Pang 2021).

### 2.5 Partially specified Boolean networks

A shortcoming of standard Boolean networks is the necessity to fully define all update functions before any analysis can take place. To address this problem, the notion of *partially specified Boolean network* (PSBN) was recently introduced (Beneš *et al*. 2023). In a PSBN E, the update functions are defined by Boolean expressions that incorporate *uninterpreted function symbols*. Each uninterpreted function is a stand in for an unknown (fixed but arbitrary) biological process. A standard BN is then obtained by assigning a concrete Boolean function to each uninterpreted symbol. Such assignment is called an *interpretation I*, and the resulting standard BN is denoted E(I). Naturally, a single PSBN can have many interpretations, meaning it encodes an ensemble of BNs consistent with its partially specified update functions. Similar to the case of admissible perturbations, this set of all interpretations (denoted I(E)) can be in practice subject to additional criteria based on known biological assumptions (e.g. essentiality, monotonicity, or canalization of specific regulators). Phenotype control is then defined for PSBNs on a per-interpretation basis: Instead of computing perturbations that universally control the network toward a phenotype, the result is a relation which assigns each perturbation *Q* a set of controlled PSBN interpretations $I_{Q}$. To assess the quality of a specific perturbation *Q* we then use its *robustness* defined as $\rho (Q)=\frac{\left|I_{Q}\right|}{\left|I(E)\right|}$. Robustness can be also understood as the likelihood that a randomly selected interpretation is controlled by the perturbation *Q*.

## 3 Methods

### 3.1 Control-guided refinement workflow

**Figure 2:**
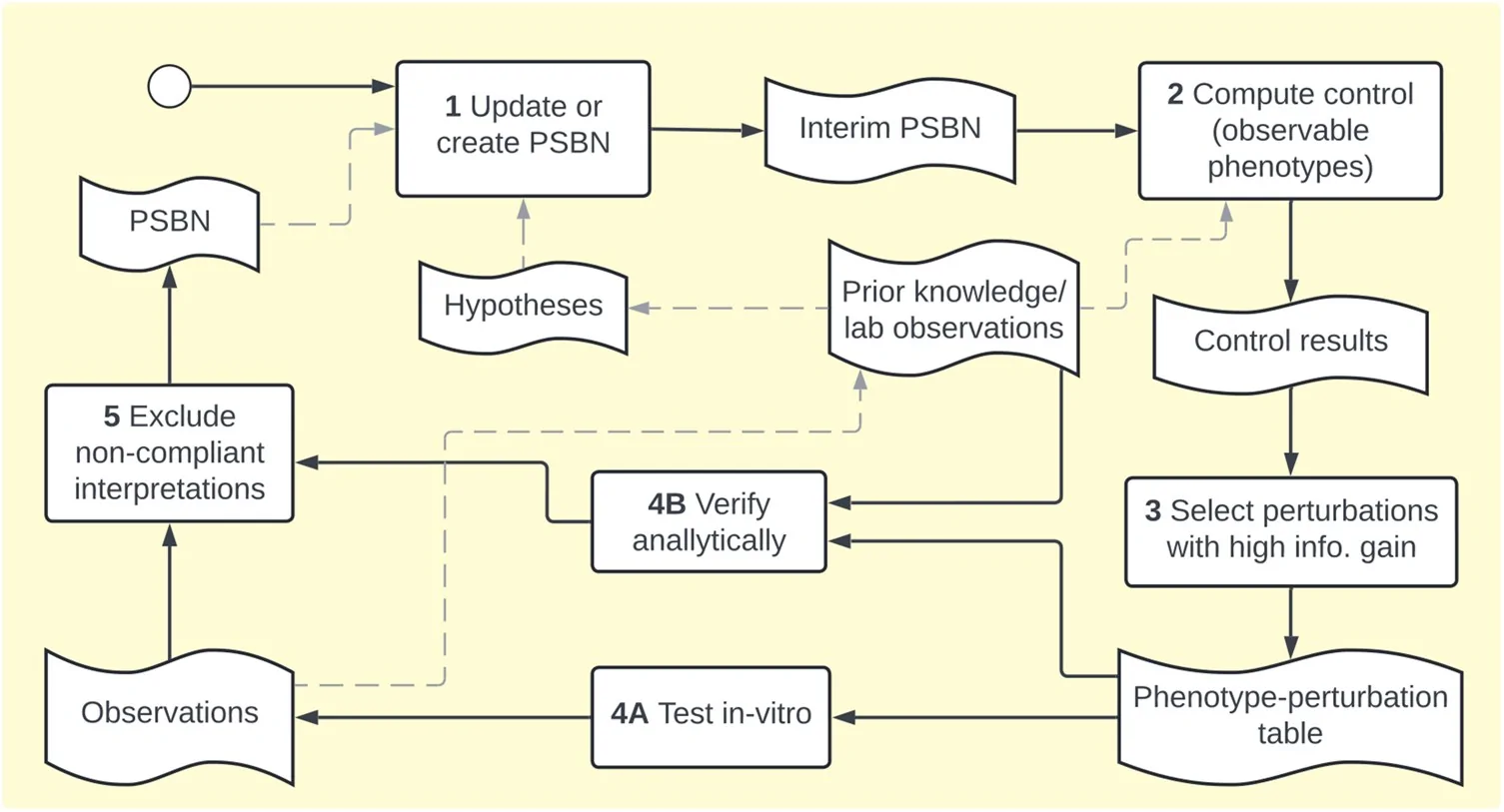
Control-guided refinement of partially specified Boolean networks. The numbered rectangle boxes represent the steps of the workflow, while the tape-shaped boxes represent the artifacts obtained from the conducted steps. The weaker arrows indicate optional paths.

In Fig. 2, we outline our *control-guided model refinement workflow*. Its goal is to select and apply perturbation experiments with high potential for refining the PSBN.

### 3.2 Initial PSBN model

We start with an initial PSBN model (Step 1; Fig. 2) and its phenotypes. Such PSBN can be constructed directly from prior knowledge and known assumptions about biological behavior, or it can be an extension of some existing model relying on new information (e.g. newly discovered regulations or pathways).

### 3.3 Screening admissible perturbations

We analyze the feasible perturbations w.r.t. known phenotypes of the PSBN (Step 2; Fig. 2). In therapy design, we would seek perturbations of high robustness achieving our phenotype of interest. For refinement, perfect robustness (i.e. ρ(Q)=1.0) is *not* desirable as it means the perturbed interpretations are indistinguishable through phenotype observations. Similarly, if two perturbations achieve equivalent phenotypes across all interpretations, only one needs to be considered further.

### 3.4 Selection of perturbations using information-gain

While the initial screening can significantly reduce the space of admissible perturbations, it likely still remains too large to explore *in vitro*. We therefore prioritize perturbations using a *normalized mutual information score* (NMIS; Kent 1983), quantifying how well is the outcome of a perturbation explained by a specific unknown *feature* of the PSBN (Step 3; Fig. 2).

To compute NMIS, we observe that interpretations I can be partitioned into equivalence classes $I_{1},\ldots ,I_{k}$ based on their outcomes, with each class consisting of interpretations that are indistinguishable by any admissible perturbation. Each class is assigned a set of *behavior labels* and *feature values*.

Here, behavior labels correspond to the phenotypes observed when subject to individual perturbations (collectively, we call these the *behavior pattern* of $I_{i}$). Similarly, *feature values* are based on selected properties of the underlying class of networks $I_{i}$. These features are derived from the unknown elements of the PSBN, such as the uninterpreted update functions or properties of regulations (essentiality, monotonicity, canalization, etc.). Specific examples are discussed later in the Results section.

We use NMIS to express the mutual information between the behavior labels achieved by a perturbation *Q* and the values given by a particular network feature, quantifying how well the network feature predicts the outcome of perturbation *Q*.

High NMIS for a feature-perturbation pair indicates that observing the perturbed phenotypes should (with high likelihood) either confirm or refute that network feature. The perturbation with the highest NMIS (for some network feature) is selected for *in vitro* testing (Step 4A; Fig. 2). Alternatively, prior knowledge can be introduced to assert the expected phenotypes of the model under perturbation (Step 4B; Fig. 2).

Finally, model instances that do not conform to the expected behavior are excluded (Step 5; Fig. 2), allowing us to restart the workflow with an updated PSBN.

### 3.5 Symbolic control algorithms

A key element of the workflow is the computation of phenotypes under perturbation across PSBN interpretations. PSBNs are subject to exponential explosion both in the number of states and interpretations. Furthermore, admissible perturbations add another layer of complexity to the overall problem.

To that end, we use a symbolic encoding based on binary decision diagrams (BDDs), allowing concise representation of large sets (or relations) of Boolean vectors. Because states, interpretations, and perturbations can be all encoded using Boolean vectors (Section 2.2, available as supplementary data at *Bioinformatics* online), BDDs achieve substantial speedup compared to brute force enumeration (Beneš *et al*. 2022). We previously explored the basis of this control approach in Beneš *et al*. (2023a); here, we present an extended version allowing for control toward a particular phenotype-attractor relationship (oscillation in particular).

In our presentation (Algorithm 1), we describe an algorithm that operates on a fixed instance of a perturbed STG[Q](E(I)) (E(I) being the BN corresponding to interpretation *I* of PSBN E). Its input is the phenotype Φ (a symbolic set of states) and the desired relationship {S,A,O}. The output is a Boolean value indicating whether the desired relationship is valid across the whole network (i.e. across all attractors of STG[Q](E(I))).

In our symbolic encoding, we can represent STG[Q](E(I)) for all combinations of I∈I and Q∈Q as a single, unified symbolic structure. Consequently, we can execute the algorithm as described here, but across all pairs of (I,Q) as a single symbolic computation, yielding a relation of (I,Q) pairs that achieve the desired control (Section 2.2, available as supplementary data at *Bioinformatics* online). This symbolic relation is then the basis for the computation of robustness and NMIS in the refinement workflow.

**Algorithm 1** Phenotype Control Algorithms 1: **function**  IsControlled($\Phi \subseteq B^{n}$, osc  ∈B)

2:  phenotype_space  ⇐  Bwd(Φ) **if**  osc  **else**  Φ

3:  phenotype_trap n ⇐  Trap(phenotype_space)

4:  non_phenotype_trap  ⇐  Trap($B^{n}\setminus$phenotype_trap)

5:  **return** non_phenotype_trap =∅

6: **end function** 

7: **function**  IsPhenotypeControl($\Phi \subseteq B^{n}$,  type  ∈{S,A,O})

8:  **if**  type=S  **then** 

9:   **return**  IsControlled(Φ, false)

10:  **if**  type=A  **then** 

11:   **return**  IsControlled($B^{n}\setminus \Phi$, false)

12:  in_phen n ⇐  IsControlled(Φ,  true)

13:  out_phen  ⇐  IsControlled($B^{n}\setminus \Phi$,  true)

14:  **return** in_phen ∧ out_phen

15: **end function** 

To explain the idea behind Algorithm 1, first we introduce Bwd(X) as the function computing all states backwards-reachable from the set *X*, and Trap(X) as the function computing the largest subset of *X* that has no outgoing transitions (i.e. largest subset of *X* that is a *trap set*). Observe that for any attractor A⊆X, we also have A⊆Trap(X). Similarly, for any attractor A∩X=∅, we have A⊆Trap(Bwd(X)). Finally, if A∩X=∅, then A∩Bwd(X)=∅ as well (an attractor cannot *reach X*, unless it *intersects X*).

Then, in the case of type=S, i.e. stabilization in Φ, we first compute Trap(Φ), retaining all attractors A⊆Φ. We then investigate $T\mathrm{rap}(B^{n}\setminus T\mathrm{rap}(\Phi ))$, which retains all attractors avoiding or oscillating through Φ. If this set is empty, stabilization in Φ is guaranteed. For avoidance, the relationship is symmetric when using $\Phi^{\prime }=B^{n}\setminus \Phi$. Note that at no point, we needed to enumerate all attractors. Instead, we focus on the largest trap sets that still contain all relevant attractors.

For oscillation, per our previous discussion of Trap(Bwd(Φ)), Iscontrolled(Φ,true) is true if all attractors either stabilize or oscillate through Φ. Symmetrically, $\mathrm{Iscontrolled}(B^{n}\setminus \Phi ,\mathrm{true})$ indicates whether all attractors stabilize or oscillate through $B^{n}\setminus \Phi$. If both of these are true, the only logical conclusion is that all attractors oscillate through Φ (and symmetrically through $B^{n}\setminus \Phi$).

## 4 Results

We demonstrate our method on two biological case studies: First is a small PSBN (12 variables, 16 interactions) reflecting current knowledge of FGFR3-MAPK signaling, isolated from other interactions within the cell. As this process has not been captured by a Boolean network before, the model incorporates four uncertain interactions and three uninterpreted functions that we aim to identify using control-guided refinement.

Subsequently, second case study considers an embedding of this isolated mechanism into the larger model of Grieco *et al*. (2013) (54 variables, 104 interactions). Here, two uninterpreted functions and six uncertain interactions arise due to the newly introduced FGFR3-MAPK signaling component. Using our method, we identify tentative perturbations suitable for identification of these uncertain model elements.

Case studies were conducted using AEON.py (Beneš *et al*. 2022), now extended with the new algorithms described in the previous section. The full source code, models, and Jupyter notebooks used for the experiments along with the results are available on Zenodo https://doi.org/10.5281/zenodo.16886813.

### 4.1 Isolated FGFR3–MAPK model

Based on our previous research (Hajnal *et al*. 2016), we construct a PSBN model of the FGFR3-MAPK signaling pathway focusing on signal activation, incorporating active forms of FGFR3, FRS2, SHIP2, SPRY, and ERK proteins. The PSBN model consists of 12 variables and 16 regulations (exact definition in Section 3.1, available as supplementary data at *Bioinformatics* online). The model contains a single input, FGFR3_stimulus, whose activity represents activation of the pathway via suitable FGF ligands. The update functions of FGFR3 and FRS2 are partially unknown, as the combined effect of incoming regulations is not yet well understood (Foldynova-Trantirkova *et al*. 2012, Kumar *et al*. 2023).

Since ERK (MAPK) is the primary target of FGFR3 downstream signaling, we consider {ERK↦1} to be the phenotype-determining *trait* of this model. We refer to the *phenotype* corresponding to this trait simply as the “ERK phenotype.” Each attractor then either stabilizes in the ERK phenotype, avoids it, or oscillates through it, resulting in ERK *stabilization*, ERK *avoidance*, and ERK *oscillation*.

The initial PSBN admits 888 interpretations; our goal is to reduce this number via perturbation-guided refinement. For simplicity, we rely on experimental observations already available in the literature, but use perturbation-guided refinement to select these observations.

#### 4.1.1 Initial perturbation screening

First, to gain insight into the behavior of our PSBN, we explore phenotypes of the unperturbed model (denoted Ø) and 21 different single-variable perturbations. Here, ERK is excluded as it is the phenotypic trait, and FGFR3_stimulus = 1 is excluded because it is already set to 1 in the unperturbed model.

The results are summarized in Table 2 and serve as an initial filter of viable perturbation candidates. Out of the 21 perturbations, only 6 do not achieve a singular phenotype across all PSBN interpretations. Conversely, for the remaining 15 perturbations, their robustness in all phenotypes is either 1 or 0. This means the perturbation results in a PSBN whose interpretations *cannot be* distinguished using observations of its phenotypes, making it useless for model refinement.

**Table 2:** Phenotype control of the isolated FGFR3 pathway model.^a^

Phenotype	Perturbations	ρ
ERK stabil.	FRS2 = 1, GRB2 = 1, MAPK3K1_3 = 1, MEK1_2 = 1, RAF =1, RAS =1, SOS =1 .	1
	FGFR3 = 1	0.94
	SPRY =0,Ø, SHIP2 = 1, SPRY =1, SHIP2 = 0	≤ 0.637
ERK avoid.	FGFR3 = 0, FRS2 = 0, GRB2 = 0, MAPK3K1_3 = 0, MEK1_2 = 0, RAF =0, RAS =0, SOS =0 .	1
	FGFR3_st. =0	0.667
	SPRY =1	0.017
ERK oscil.	SHIP2 = 0	0.41
	Ø, SHIP2 = 1	0.374
	SPRY =0	0.363
	SPRY =1, FGFR3 = 1, FGFR3_st. =0	≤ 0.357

a The table shows the distinct perturbations of size up to one ($2^{\text{nd}}$ column) that control the model toward the phenotypes stated in the $1^{\text{st}}$ column. The symbol Ø denotes the unperturbed model. The robustness in each case is computed based on all interpretations of the initial PSBN ($3^{\text{rd}}$ column). If a perturbation is not shown for a specific phenotype, it means it has a robustness of zero (the phenotype is impossible given this perturbation).

Note that we also tested perturbations of size two, but no such perturbation reached meaningfully different robustness value. As such, we focus on single-variable perturbations. We also performed a more in-depth exploration of phenotypes attained by the 6 perturbations of interest (Fig. 10, available as supplementary data at *Bioinformatics* online) and uncovered that SHIP2 = 1 does not in fact alter the phenotype repertoire compared to the unperturbed model. Thus, we exclude it from consideration.

#### 4.1.2 Feature-based perturbation selection

To select the most relevant perturbations out of the 5 remaining, we consider which features of the PSBN best explain the perturbation outcomes. First, the candidate perturbations classify the 888 PSBN interpretations into 16 equivalence classes based on the phenotypes of the perturbed model (i.e. ERK stabilization, avoidance, or oscillation). We then assign each class a collection of feature values, corresponding to concrete properties of the candidate BNs. Each such feature can be either held universally by *all* members of the class (denoted ∀), or existentially (denoted ∃) by *some* members of the class (naturally, if a feature is universally held in a class, it is by definition also held existentially). Here, the specific set of features consists of (i) essentiality of the five uncertain regulations; (ii) individual interpretations of the network’s partially unknown update functions.

For every perturbation-feature pair, we compute the NMIS between the perturbation outcome and the feature. This score indicates which features correctly predict phenotypes of the perturbed model. The results of this analysis are presented in Table 3. Here, the most prominent perturbation is FGFR3_stimulus = 0, where the phenotypes are perfectly predicted by the choice of update function in FGFR3 (NMIS is 1.0). The second most prominent feature is the regulation SPRY→FRS2 when subject to perturbation SHIP2 = 0 (NMIS is 0.855). Both SHIP2 and SPRY are considered uncertain regulators of FRS2 in our PSBN, which is the second variable with a partially specified update function in our model. Consequently, this indicates that the interplay of these two regulators is the most important for determining the update function of FRS2.

**Table 3:** Mutual information score of interpretation features.^a^

	Pert.		Feature	NMIS
	Ø	$F_{1}$	∀ SPRY→FRS2	0.364
		$F_{2}$	∃ SPRY→FRS2	0.279
			∀ SPRY→SHIP2	0.279
$Q_{1}$	SPRY = 0	$F_{3}$	∃ FGFR3 = FGFR3_st. ∧ ¬ GRB2	0.529
		$F_{4}$	∃ GRB2→FGFR3	0.293
			∃ FRS2 = FGFR3_st.	0.293
$Q_{2}$	SHIP2 = 0	$F_{2}$	∃ SPRY→FRS2	0.855
		$F_{5}$	∀ FRS2 = FGFR3 ∧ SHIP2	0.735
			∃ FRS2 = FGFR3 ∧ SHIP2	0.735
$Q_{3}$	FGFR3_st.=0	$F_{6}$	∀ FGFR3 = ¬ GRB2 ∨ FGFR3_st.	1.000
			∃ FGFR3 = ¬ GRB2 ∨ FGFR3_st.	1.000
$Q_{4}$	SPRY = 1	$F_{1}$	∀ SPRY→FRS2	0.485
		$F_{7}$	∃ FRS2 = FGFR3 ∧ ¬ SPRY	0.437
$Q_{5}$	FGFR3 = 1	$F_{8}$	∃ SHIP2→FRS2	0.562
		$F_{1}$	∀ SPRY→FRS2	0.562
		$F_{2}$	∃ SPRY→FRS2	0.442
			∀ SHIP2→FRS2	0.442

a NMIS of the three best performing features (four in case of equal scores) per each labelling (assessment of phenotypes under perturbation). We then consider only non-redundant features (e.g. an existential feature is redundant if its universal variant has an equal or higher NMIS); these are assigned names $F_{1},\ldots ,F_{8}$.

#### 4.1.3 Model refinement

Based on our results in Table 3, we consider FGFR3_stimulus = 0 ($Q_{3}$) and SHIP2 = 0 ($Q_{2}$) to be the most relevant for the identification of a concrete model refinement. We performed a literature search to identify plausible experimental results for these two most relevant perturbations, leading to the following assumptions: First, based on Foldynova-Trantirkova *et al*. (2012), we expect FGFR3_stimulus to be *necessary* to achieve ERK stabilization or oscillation in this isolated model. Second, based on Fafilek *et al*. (2018), we observe that SHIP2 = 0 knock-out causes down-regulation of ERK activity.

To put these assumptions into context, we prepared Table 4, which partitions the PSBN interpretations into equivalence classes based on the effects of our five chosen perturbations on its phenotypes. Within this table, we can easily identify that only 9 equivalence classes (namely 1,2,4,5,7,9,12,14,16) adhere to our first assumption, completely eliminating ERK activity in response to FGFR3_stimulus = 0 ($Q_{3}$) perturbation. Out of these nine options, the second assumption allows to select rows 5, 12, and 14, as these are the only cases where the activity of ERK is plausibly down-regulated compared to the unperturbed network in response to SHIP2 = 0 knock-out ($Q_{2}$), resulting in 20/888 candidate networks.

**Table 4:** Behavior of the isolated FGFR3 pathway under perturbations.^a^

		**ERK under perturbation**						∃A→B			∀A=ex.	
**#**	∣I∣	**Ø**	$Q_{1}$	$Q_{2}$	$Q_{3}$	$Q_{4}$	$Q_{5}$	$F_{2}$	$F_{4}$	$F_{8}$	$F_{5}$	$F_{6}$
**1**	277	⇄	⇄	⇄	0	⇄	1	1	1	1	0	0
**2**	259	1	1	1	0	1	1	0	0	0	0	0
**3**	259	1	1	1	⇄	1	1	0	1	0	0	1
**4**	22	⇄	⇄	⇄	0	⇄	⇄	1	1	0	0	0
**5**	18	1	1	⇄	0	1	1	1	0	1	0	0
**6**	18	1	1	⇄	⇄	1	1	1	1	1	0	1
**7**	9	⇄	⇄	⇄	0	0	⇄	1	1	0	0	0
**8**	9	⇄	⇄	⇄	⇄	⇄	⇄	1	1	0	0	1
**9**	4	⇄	1	⇄	0	⇄	⇄	1	0	0	0	0
**10**	4	⇄	1	⇄	⇄	⇄	⇄	1	1	0	0	1
**11**	4	⇄	⇄	⇄	⇄	0	⇄	1	1	0	0	1
**12**	1	⇄	⇄	0	0	⇄	1	0	1	1	1	0
**13**	1	⇄	1	⇄	⇄	0	⇄	1	1	0	0	1
**14**	1	1	1	0	0	1	1	0	0	1	1	0
**15**	1	1	1	0	⇄	1	1	0	1	1	1	1
**16**	1	⇄	1	⇄	0	0	⇄	1	0	0	0	0

a Each row corresponds to a class of interpretations characterized by their perturbation response. Here, # is the row index and |I| is size of the interpretations class. Then, we describe ERK phenotypes achieved under specific perturbations, as listed in Table 3 (phenotypes represent ERK stabilization (1), avoidance 0, or oscillation (⇄)). The last two columns represent selected model features (also listed in Table 3) representing guaranteed presence of regulations, and guaranteed update function specification. Rows highlighted in gray allow non-trivial ERK activity even with FGFR3_st.=0, violating our first assumption. The three light grey rows are the rows which satisfy both of our control-guided assumptions.

To disambiguate between rows 5, 12, and 14, we would have to quantify the effects observed by Fafilek *et al*. (2018). However, based on the fact that Fafilek *et al*. (2018) do not report ERK to be completely inactive, we can safely eliminate rows 12 and 14 (where ERK phenotype drops to 0). Furthermore, considering the fact that ERK is known to oscillate (Raina *et al*. 2022), we prefer row 5 as the most likely option.

Interestingly, the 18 networks left in the class at row 5 are completely indistinguishable both in terms of their unperturbed phenotypes and their phenotype response to single-variable perturbations. More importantly, in these 18 networks, it universally holds that GRB2 does *not* regulate FGFR3, but SHIP2 always regulates FRS2 (other regulations remain undetermined).

### 4.2 Extended FGFR3–MAPK model

We consider the fully specified model of Grieco *et al*. (2013) that puts FGFR3-MAPK signaling into a broader context of protein interactions having the crucial impact on cell growth/division. The model does not include several important details on the FGFR3 signal activation mechanism. To that end, based on Reactome (Milacic *et al*. 2024) and state-of-the-art knowledge (see Fafilek *et al*. 2018, 2022), we compile an extended PSBN model that embeds the isolated model into the integrative Grieco model. At the level of regulations, the embedding brings in: (i) the addition of SHIP2 affecting the FRS2 adapter phosphorylation capabilities as discussed within the isolated model, (ii) addition of the direct negative feedback from ERK to FRS2 (experimentally studied mostly in the context of chondrocytes), (iii) making all negative feedback regulations affecting FGFR3 and FRS2 non-essential.

At the level of logical rules, the update functions of FGFR3 and FRS2 are made unspecified with the only constraint that FGFR3_stimulus is left as a necessary precursor of FGFR3 activation. The chosen level of abstraction reflects the fact that biochemical mechanisms specifying how the respective regulations affecting FGFR3 and FRS2 are combined are not currently known. The model has four inputs: EGF, FGF, TGF stimuli, and DNA damage signal. To simulate an environment maximally compatible with the isolated model, we consider all inputs to be fixed to 0, with the only exception of FGFR3 stimulus. The resulting model contains 54 variables and four special output variables assigning the specific traits observed at the protein level to particular cell-level phenotypes—apoptosis (A), growth arrest (GA), no decision (ND), and proliferation (P). These variables are employed to define the cardinal (cell-level) phenotypes based on stabilization of one of these variables at value 1. The complete model as well as specification of phenotypes is included in Section 3.2, available as supplementary data at *Bioinformatics* online.

The goal of the analysis is to apply control-guided refinement to identify perturbations that can reduce the set of possible interpretations of the extended PSBN. We consider all experimental observations and assumptions applied in the analysis of the isolated model while reflecting the prior knowledge of the cell behavior based on Grieco *et al*. (2013), Vaqué *et al*. (2014), and Stanislaus *et al*. (2012).

First, we compute control for all phenotypes (step (2)), restricting ourselves to perturbations up to size one. The relevant results are shown in Table 5 while the full results can be found in Section 3.3, available as supplementary data at *Bioinformatics* online. We observe that perturbations can achieve all considered cell-level phenotypes including long-term dynamics oscillating among all of them (denoted “⇄  ∀”). We can also observe the correlation between the ERK activity and the proliferation phenotype—in particular, the stabilization of {ERK↦1} is required for the proliferation phenotype while apoptosis phenotype implies avoidance of {ERK↦1}.

**Table 5:** Phenotype control of extended FGFR3–MAPK model.^a^

Φ	Perturbations	ERK	ρ for I	ρ for I
A	*ATM =1, CREB = 0, DNA_dmg = 1, DUSP1 = 0 ,* *TAK1 = 1, TAOK = 1, TGFBR = 1, TGFBR_st.=1*	0	1	1
	**PLCG =0**	0	0.80	1
	AP1 = 1, ERK =0, GADD45 = 1, JNK =1, JUN =1, MEK1_2 = 0, MTK1 = 1, PPP2CA =1, SMAD =1, p38 = 1, p53 = 1, SHIP2 = 1, SHIP2 = 0, PKC =1, Ø, SPRY, RSK =0, PKC =0, SPRY =1 .	0	< 0.6	< 0.5
GA	BCL2 = 1, FOXO3 = 0, JNK =0	0	0.61	0
	ERK =1, MEK1_2 = 0, p21 = 1	1	< 0.3	0
ND	AKT =1, PTEN =0; p38 = 0 .	0	0.61	0
	*MAP3K1_3 = 0, MSK = 0, MYC = 0, RAS = 0*	*	0.50	1
	AKT =0, *FGFR3 = 0*, GAB1 = 0, RSK =1, SOS =0, *FGFR3_stimulus =0*, p53 = 0, p70 = 0, PDK1 = 0, PI3K =0, *PKC =1*, PLCG =0, PTEN =1 .	*	< 0.3	< 0.2
P	**ERK =1**, *MEK1_2 = 1*	1	0.3	1
	*RAF =1*	1	0.19	1
	GAB1 = 1, GADD45 = 0, MAX =0, MDM2 = 1, MTK1 = 0, p14 = 0, p53 = 0, PDK1 = 1, PI3K =1 .	1	< 0.1	0
⇄ ∀	***Ø***	⇄	0.28	1

a The first column shows the phenotype manifested by the model under perturbations listed in the second column. Perturbations in blue font are working in the original Grieco *et al*. (2013) model. Perturbations highlighted in light grey are the reference perturbations we used for the model refinement. The third column displays the long-term ERK activity, and the last columns show the robustness of respective perturbations. The symbol “*” denotes that we observe ambiguous ERK behavior under the given perturbations.

The results highlighted in italics correspond with the perturbations reported in Grieco *et al*. (2013) (see Section 3.2, available as supplementary data at *Bioinformatics* online). Additionally, the results include several perturbations not considered before. For example, the model predicts that the perturbation PLCG = 0 leads to apoptosis stabilization. This observation is well-aligned with Vaqué *et al*. (2014); Stanislaus *et al*. (2012) reporting that PLCG is a driver for cell proliferation while its inhibition can lead to apoptosis. It gives a strong evidence for selecting the predicted perturbation for the model refinement. Based on these findings, we can conclude the perturbations ERK = 1 (achieving proliferation) and PLCG = 0 (achieving apoptosis) are strongly affecting the long-term behavior of the model. We use these insights to complete the first iteration of the refinement procedure. We have achieved the reduction from 556 480 to 53 532 interpretations.

Next, we recompute the robustness values for the reduced set of interpretations satisfying the observations discussed in the previous paragraph used as the reference set I (the last column of Table 5). Subsequently, we select perturbations that partition interpretations into distinct behavior patterns (see also Section 3.3, available as supplementary data at *Bioinformatics* online). Based on these results we identify the admissible perturbations that can be considered for *in vitro* perturbation experiments design leading to potential exclusion of incompliant interpretations of the PSBN (Table 6).

**Table 6:** Behavior of interpretations under perturbations of extended MAPK model.

#	∣I∣	FGFR3 = 0	FRS2 = 0	PKC =1	SHIP2 = 0	SHIP2 = 1
**1**	10752	A	⇄∀	A	A	⇄∀
**2**	10612	⇄∀	⇄∀	⇄∀	⇄∀	A
**3**	8192	⇄∀	⇄∀	⇄∀	⇄∀	⇄∀
**4**	3848	⇄∀	A ∣ ND ∣ P	⇄∀	ND ∣ P	A
**5**	3158	⇄∀	A ∣ ND ∣ P	⇄∀	⇄∀	A
**6**	2880	A	A ∣ ND ∣ P	A	A	⇄∀
**7**	2430	⇄∀	A ∣ ND ∣ P	⇄∀	ND ∣ P	⇄∀
**8**	2160	A	A ∣ ND ∣ P	A	A ∣ ND ∣ P	⇄∀
**9**	1980	⇄∀	A ∣ ND ∣ P	⇄∀	⇄∀	⇄∀
**10**	1666	ND	⇄∀	ND	⇄∀	A
**11**	1666	⇄∀	⇄∀	ND	⇄∀	A
**12**	1280	ND	⇄∀	ND	⇄∀	⇄∀
**13**	1280	⇄∀	⇄∀	ND	⇄∀	⇄∀
**14**	624	ND	A ∣ ND ∣ P	⇄∀	ND ∣ P	A
**15**	390	ND	A ∣ ND ∣ P	⇄∀	ND ∣ P	⇄∀
**16**	288	ND	A ∣ ND ∣ P	⇄∀	⇄∀	A
**17**	180	ND	A ∣ ND ∣ P	⇄∀	⇄∀	⇄∀
**18**	43	ND	A ∣ ND ∣ P	⇄∀	A ∣ ND ∣ P	A
**19**	43	⇄∀	A ∣ ND ∣ P	⇄∀	A ∣ ND ∣ P	A
**20**	30	⇄∀	A ∣ ND ∣ P	⇄∀	A ∣ ND ∣ P	⇄∀
**21**	30	ND	A ∣ ND ∣ P	⇄∀	A ∣ ND ∣ P	⇄∀

a The first two columns denote the index and the size of the interpretation set, while the next five columns denote the behavior of the interpretations under the selected perturbations. The ∣ separates possible bi-stability outcome while ⇄∀ denotes oscillation between all model phenotypes.

We see that some perturbations can produce bi- or tri-stable behavior. However, this appears to be critical only for SHIP2 = 0 perturbation, where we might struggle to differentiate between overlapping bi-stability or tri-stability outcome. In that case we might not be able to establish a single behavior class (e.g. rows #14 and #18 differ only in SHIP2 = 0—if relevant perturbation experiments do not exhibit (stable) apoptosis, we cannot distinguish between the two classes of interpretations).

## 5 Conclusion

In general, we have shown that *our approach is able to reduce the number of interpretations* of the initial PSBN. The method has the capability to identify potential perturbation experiments providing *significant information gain toward further reduction* of possible interpretations.

With the isolated model, we have targeted the FGFR3-ERK pathway incorporating non-trivial negative feedback mechanisms. Enabling the PSBN phenotype control *to handle oscillatory phenotypes allowed us to confirm state-of-the-art experimental observations with the qualitative modelling framework*. In particular, we have computationally confirmed novel experimental insights on effects of SHIP2 observed in Fafilek *et al*. (2018)—in our model SHIP2 loss of function leads to observable activity (here oscillation) of ERK.

By embedding the isolated FGFR3 mechanism into the larger model targeting the control of growth-related phenotypes, we have shown the ability of our method to infer biologically relevant insights confirming that the activation profiles of ERK are tightly coupled with proliferation and apoptosis. Again, the introduction of oscillatory phenotypes within the PSBN control framework has been crucial in identifying perturbations having high information gain toward elimination of the existing knowledge gaps.

From the performance point of view, most of the computations were completed in reasonable time, with the extended model needing up to 10 h (see Table 11, available as supplementary data at *Bioinformatics* online) for some of the most complex instances of oscillatory phenotypes. The most crucial bottleneck for our method is arity of the unknown update functions, as in the worst case, it can lead to guaranteed exponential blow-up even in the symbolic BDD representation that we use. For future work, we aim to combine our approach with other model analysis techniques (e.g. model checking or model classification; Beneš *et al*. 2023a).

## Supplementary Material

btag275_Supplementary_Data
