# Correction to: Control-guided refinement of partially specified Boolean networks: applications to RTK signaling

**DOI:** 10.1093/bioinformatics/btag773

**Published:** 2026-10-20

This is a correction to: Eva Šmijáková, Luboš Brim, Samuel Pastva, David Šafránek, Control-guided refinement of partially specified Boolean networks: applications to RTK signaling, *Bioinformatics*, Volume 42, Issue 6, June 2026, btag275, https://doi.org/10.1093/bioinformatics/btag275

In the originally published version of this manuscript, Figures 1 and 2 were erroneously switched. This error has been corrected.

Additionally, the ‘Example’ column in Table 1 contained erroneous characters.

The corrected Table 1 reads:

**Table 1:** Phenotype related terms.^a^

Concept	Real-world	Example	Network	Example
Character	Properties of interest	Presence of FRS2 tyrosine residue phosphorylation	Set of BN variables	{FRS2}
Trait	Observation of properties	FRS2 tyrosine residue is not phosphorylated	Character valuation; alternatively a subspace	{FRS2 → 0} ⋆0⋆
Phenotype	Observation combination	FRS2 or ERK is phosphorylated	Combination of traits	⋆ 1 ⋆ ∪ ⋆ ⋆ 1

a The table explaining the notions of character, trait, and phenotype in the context of real-world concepts in Boolean network models. The considered example is a small excerpt from the FGFR3-MAPK pathway in our case study.

