# Supplementary material for: Control-guided refinement of partially specified Boolean networks: applications to RTK signaling
Source: Bioinformatics. 2026 May 24;42(6):btag275. doi: 10.1093/bioinformatics/btag275 (PMC13303288; doi:10.1093/bioinformatics/btag275)
Supplement: btag275_Supplementary_Data [file btag275_supplementary_data.zip › notation.pdf]

Notation

	**Boolean algebra related symbols**
b	Boolean value (true or false)
$\mathbb{B}$	Set of Boolean values (true or false)
$\star$	Undefined Boolean value (might be true or false)
f	A function
$\mathbb{B}_\star$	Set of Boolean values including the undefined value
$x[i \mapsto b]$	A vector x with the i-th element replaced by b
	**Networks - common**
n	Size of a network (number of its nodes – variables)
$\mathbb{E}$	Edges of a network
$x, s, t, \ldots$	States of a network
$\mathbb{U}$	All states of a network
S	Subspace of a network
$\mathcal{F}$	Boolean network
STG	Asynchronous state transition graph
`var`	A font for variable names
`var1`$\rightarrow$`var2`	Influence of `var1` on `var2` is observable in STG
A	An attractor of a (partially specified) Boolean network
$\mathbb{A}$	Set of all attractors of a (partially specified) Boolean network
$\mathcal{U}$	An observed character of a network
$\mathcal{T}$	A trait of a network
Φ	A phenotype of a network
	**Partially specified Boolean networks**
$\mathcal{E}$	Partially specified Boolean network, a set of all function symbols
g	A function symbol of a partially specified Boolean network
$\mathbb{G}$	A set of all function symbols of a partially specified Boolean network
E	An expression of a partially specified Boolean network
I	An interpretation of a partially specified Boolean network
$\mathcal{I}$	A set of interpretations
$\mathbb{I}$	Set of all interpretations of a partially specified Boolean network
	**Control**
Q	A perturbation of a network
$\mathbb{Q}$	A set of all admissible perturbations
ρ	Robustness of a perturbation towards considered set of interpretations
