# Supplementary material for: Control-guided refinement of partially specified Boolean networks: applications to RTK signaling
Source: Bioinformatics. 2026 May 24;42(6):btag275. doi: 10.1093/bioinformatics/btag275 (PMC13303288; doi:10.1093/bioinformatics/btag275)
Supplement: btag275_Supplementary_Data [file btag275_supplementary_data.zip › appendix.pdf]

Control-Guided Refinement of Partially Specified Boolean Networks: Applications to RTK Signalling (Supplementary Material)

This document supplements the main paper by providing additional details, clarification, and results. Each section is a self-contained extended version of the respective section from the main text. Section 1 offers an expanded exposition of the preliminaries introduced in the main text, including more in-depth explanations and illustrative examples. Section 2 builds on Methods from the main text. Here, Subsection 2.1 presents the control workflow for therapy design and model refinement in greater detail, with a particular focus on the analytical methods used for control-guided model refinement. Subsection 2.2 describes the algorithms used in the paper, including the symbolic state space exploration techniques that enable efficient analysis of partially specified Boolean networks and examples of algorithm progressions. Finally, Section 3 presents additional results, including the results computed using original model from [16] and more detailed results of other experiments.

1 Preliminaries: Detailed Exposition

This section presents a formal introduction to the topics essential for this work, including regulatory networks, Boolean networks, partially specified Boolean networks, the notion of permanent perturbation, phenotype control and perturbation robustness. All the notation introduced here is also overviewed in the supplement material *Notation*.

Notation. We write $\mathbb{B}$ and $\mathbb{B}_\star$ to denote the domains $\{0,1\}$ and $\{0,1,\star\}$. Here, $\star$ is a *free* value (i.e., neither 1 nor 0). We write $\mathbb{B}^n$ to denote the set of all n-element vectors over $\mathbb{B}$. In the following, such n refers to the *size* of a Boolean network, while x represents its state (a configuration). For each $x \in \mathbb{B}^n$, x_i then denotes the i-th element of x. Finally, for $x \in \mathbb{B}^n$, index $i \in [1,n]$, and a Boolean value $b \in \mathbb{B}$, expression $x[i \mapsto b]$ denotes a substitution of the i-th element in x for the value b. Formally, the result is $x' = x[i \mapsto b]$ s.t. $x'_j = b$ for $j = i$, and $x'_j = x_j$ otherwise.

1.1 Regulatory networks

Regulatory networks (RNs) play a pivotal role in understanding the intricate dynamics of biological system components (genes, proteins, and other regulatory elements). We consider its measurable property, e.g., phosphorylation, expression level of mRNA, or some other activity, but for a shorthand usually only the high level component name is stated in the RN model. These components interact in semantically various ways (promoter binding, expression, etc.) which details are however also abstracted in the RN.

Formally, a regulatory network can be defined as a mathematical abstraction representing the interactions among molecular components within a biological system.

Let $\mathcal{G} = (\mathbb{U}, \mathbb{E})$ denote a directed graph representing the regulatory network, where $\mathbb{U}$ is the set of states representing the molecular components and $\mathbb{E}$ is the set of directed edges representing the regulatory interactions between the components.

Each vertex $v \in \mathbb{U}$ corresponds to a molecular component and each directed edge $(u, v) \in \mathbb{E}$ represents a regulatory influence from component u to component v. Sometimes we also label the nature of these interactions, such as activation, inhibition, or other forms of regulatory control. An example of a simple regulatory network can be found in Fig. 1.

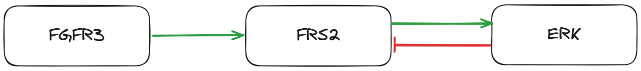

Figure 1: **An example of a simple regulatory network.** This regulatory network contains three nodes and three regulations – two activations (green) and one inhibition (red). The network is a small extract of a MAPK signaling pathway [16].

Regulatory networks are however too abstract to directly study the dynamics of the system. To this end, more specific models are needed. One of the most popular models for this purpose is the Boolean network model.

1.2 Boolean networks

Boolean networks (BNs) were introduced by Kauffman in the 1960s [19]. Kauffman represented system components as binary variables connected by logical rules. Such an abstraction facilitates the exploration of system dynamics while avoiding the computational complexity of traditional models. Since then, it has become a widely used model to study the dynamics of complex biological systems [2, 1]. Formally we define the Boolean network model as follows:

Let n be the number of system variables. A *Boolean network* is a collection $\mathcal{F} = \{f_1, \ldots, f_n\}$ with each $f_i : \mathbb{B}^n \to \mathbb{B}$ being the Boolean *update function* of the network's i-th variable.

The set $\mathbb{U} = \mathbb{B}^n$ is the network's *state space*, and the vectors $s \in \mathbb{B}^n$ are its *states*. The set of nodes (variables) is typically equal to the set of nodes of RN which served as a foundation for the BN model. Note that although the input of each BN function f_i is a full state $s \in \mathbb{B}^n$, the output of f_i does not typically depend on all network variables, but on a small subset given by the RN inbound edges to this node. Such set of variables is called regulators. Moreover, when there is a regulator j for a regulator i, such that, $\exists s \in \mathbb{B}_n : f_i(s[j \mapsto 1] \oplus s[j \mapsto 0])$ we say that the regulation $j \to i$ is *observable* (or *essential*).

Additionally, we call the members of $\mathbb{B}^n_\star$ the *subspaces* of $\mathcal{F}$. Intuitively, each subspace $S \in \mathbb{B}^n_\star$ describes a hypercube in the state space $\mathbb{B}^n$. This hyper-cube consists of states $s \in \mathbb{B}^n$ such that $s_i = S_i$ for all i where $S_i \in \mathbb{B}$. We can thus treat each subspace S as a set of states. Furthermore, to denote a specific subspace, we will often simply use a string of values from $\mathbb{B}_\star$ instead of the full vector notation (e.g. $S = 10\star$ instead of $S = (1, 0, \star)$).

1.3 Boolean network dynamics

To formally reason about the evolution of the BN instance states, we consider its asynchronous state-transition graph:

For $\mathcal{F} = \{f_1, \dots, f_n\}$, the *state-transition graph* $\mathrm{STG}(\mathcal{F}) = (\mathbb{U}, \mathbb{E})$ is a directed graph with $\mathbb{U} = \mathbb{B}^n$ and $\mathbb{E} \subseteq \mathbb{U} \times \mathbb{U}$ given as follows:

$$(s, t) \in \mathbb{E} \Leftrightarrow (s \neq t \wedge \exists i \in [1, n].\ t = s[i \mapsto f_i(s)])$$

We can write $s \to t$ whenever $(s, t) \in \mathbb{E}$. Observe that each transition within $\mathrm{STG}(\mathcal{F})$ always updates exactly one network variable. An example of a Boolean network and its state transition graph can be seen in Fig. 2.

1.3.1 Components of Boolean networks

To study the long-term behavior of a network, we use the terms *trap set* and *attractor* [15, 27]:

Let $\mathcal{F} = \{f_1, \dots, f_n\}$ be a Boolean network and $X \subseteq \mathbb{B}^n$ a set of network states. We say that X is a *trap set* when for all $x \in X$ and $y \in \mathbb{B}^n$ if we have $x \to y$ implies $y \in X$ (i.e. X cannot be escaped). We say that X is an *attractor* when X is a *minimal* trap set (if any state is removed from X, it stops being a trap set).

Equivalently, we can also define attractors as terminal strongly connected components of $\mathrm{STG}(\mathcal{F})$. We write $\mathbb{A}(\mathrm{STG}(\mathcal{F}))$ to denote the set of all attractors of $\mathcal{F}$. An example of an attractor can be seen in Subfig. 2c, where we can see a single oscillating attractor composed of states $1 * *$.

A phenotype is a set of observable characteristics of an organism, resulting from the interaction of its genetic makeup (genotype) with the environment [18]. In various cases, we might be interested in observing different sets of characteristics; e.g., in the case of a cell, we could be interested in the phosphorylation of certain proteins.

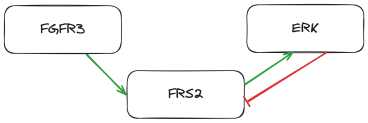

(a) Regulatory network

$$\mathtt{FGFR3} = 1$$
$$\mathtt{FRS2} = \mathtt{FGFR3} \wedge \neg \mathtt{ERK}$$
$$\mathtt{ERK} = \mathtt{FRS2}$$

(b) Boolean network

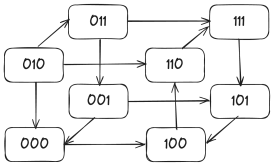

(c) BN state transition graph

Figure 2: **An example of a Boolean network and related concepts.** In Subfig. a the same RN as in 1 is displayed. This RN was used as a basis for BN defined in Subfig. b). Finally, we can depict BN's STG in Subfig. c where the labeling of states has the same order as variable order in Subfig. b.

In the context of BNs, the characteristics are represented by one or more variables. We call such a set of variables a *character*. The actual observable manifestation of this character is called a *trait* and is then an assignment of these variables as Boolean values:

A *character* is a set of observed BN variables $\mathcal{U} \subseteq [1, n]$. A *trait* $\mathcal{T}$ is then a valuation of these character variables: $\mathcal{T} : \mathcal{U} \to \mathbb{B}$.

Such character variables $\mathcal{U}$ typically correspond to the network outputs (variables that do not regulate any other network variables), but this is not required. Each trait defines a subspace $S^{\mathcal{T}} \in \mathbb{B}^n_\star$ s.t. $S^{\mathcal{T}}_i = \mathcal{T}(i)$ for $i \in \mathcal{U}$, and $S^{\mathcal{T}}_i = \star$ otherwise. With a slight abuse of notation, we simply use $\mathcal{T}$ to also mean the subspace $S^{\mathcal{T}}$ when clear from the context.

In our approach, we assume, that more than one of the characters may be observed at the same time and thus more than one trait can be manifested by the network. To express this behavior we define a *phenotype* as follows:

A *phenotype* is a set of states $\Phi \subseteq \mathbb{B}^n$ described by any combination of the BN traits. The combination of traits is composed using standard set operators.

An overview and comparison of the introduced concepts can be found in Table 1.

It has been shown that attractors are closely tied to the notion of biological phenotypes [22, 14]. Each attractor represents a possible *stable outcome* achievable within a particular $\mathbb{BN}$. As opposed to the notion of BN phenotype we have just introduced, a set of attractors the BN exhibits is an inherent property of the model given by the set of functions. In contrast, phenotypes are the modeler's choice that assign a biological meaning to an otherwise abstract set of

Concept	Real-world	Example	Network	Example
Character	A property of an interest	A phosporolation on `FRS2` tyrosine residue	A set of variables $\mathcal{U}$	{`FRS2`}
Trait	An observation	`FRS2` tyrosine residue is not phosphorilated	Valuation $T : \mathcal{U} \to \mathbb{B}$, a subspace generator	`FRS2`=0, *0*
Phenotype	A combination of observations	`FRS2` or `ERK` is phosphorylated	A combination of traits Φ	$*1* \cup **1$

Table 1: **Phenotype related terms.** The table summarizes the notions of character, trait, and phenotype in the context of real-world concepts compared to Boolean network models. The considered example of a BN is from Fig. 2.

states. Since other BN states are considered to be transient and non-observable, it makes sense to study the relationship between attractors and phenotypes.

An attractor $A \in \mathbb{A}$ may have three types of relationships toward a given phenotype Φ:

- A *stabilizes* in Φ, if $A \cap \Phi = A$ (or equivalently $A \subseteq \Phi$).
- A *avoids* Φ, if $A \setminus \Phi = A$ (or equivalently $A \cap \Phi = \emptyset$).
- A *oscillates* through Φ, if $A \cap \Phi \neq \emptyset \wedge A \setminus \Phi \neq \emptyset$.

If *all* attractors of a BN have the same relationship with a given phenotype, we can generalize the phenotype's relationship to a whole BN. For example, we can say both about the BN and the only attractor in Subfig. 2c, that it stabilizes in the phenotype 1**, and it oscillates through the phenotype **1.

1.4 Partially specified Boolean networks

A shortcoming of classical Boolean networks as defined above is that to study network dynamics, all update functions must be fully known. However, this is often not realistic for large-scale systems since we might lack the data necessary to precisely specify all the update functions.

Moreover, there might be other cases when we want to *intentionally* study multiple versions of a BN for example to consider possible mutations or differences in the studied genotypes. To address this problem, we consider the notion of *partially specified Boolean networks* (PSBNs) [4].

In a PSBN, we can use *uninterpreted functions* as stand-ins for unknown (fixed but arbitrary) parts of the network's dynamics. Each uninterpreted function is then denoted by its *symbol* (a name) and input arguments. Let n be the number of system variables, and $\mathbb{G}$ a set of *uninterpreted function symbols*. A *partially specified Boolean network* $\mathcal{E} = \{g_1, \ldots, g_n\}$ consists of expressions g_i given by the following grammar:

$$E ::= 0 \mid 1 \mid x \mid \neg E \mid E \wedge E \mid E \vee E \mid g^{(a)}(E_1, \ldots, E_a)$$

Here, x ranges over the network variables and g over the uninterpreted function symbols of $\mathbb{G}$ (superscript $a \in \mathbb{N}_0$ denotes the arity of g). Other Boolean operators (e.g. $\Rightarrow$ or $\Leftrightarrow$) can be implemented as syntactic abbreviations using $\vee$, $\wedge$, and $\neg$.

In other words, a PSBN is defined using standard Boolean constants (0 and 1), variable state propositions (x) and Boolean connectives ($\neg$, $\wedge$, $\vee$), but it can also use uninterpreted functions from $\mathbb{G}$ as a way of incorporating unknown behavior. This makes it possible to idiomatically describe systems whose dynamics are not fully known.

Note that this definition also allows zero-arity uninterpreted functions (e.g. $g^{(0)} \in \mathbb{G}$). These are effectively unknown Boolean constants. As such, they are functionally equivalent to network inputs with an unknown value. To distinguish them, we sometimes call these uninterpreted functions *logical parameters*. The framework of partially specified BNs (or BNs with parameters) then allows the translation of a function with any amount of unknown precise Boolean relationship into unary unknown symbols (logical parameters). The detailed procedure can be found in [9].

To assign meaning to a particular $\mathcal{E}$, we rely on the term *interpretation*. An interpretation I is a function that assigns each symbol from $\mathbb{G}$ a Boolean function of the corresponding arity. By substituting each $g \in \mathbb{G}$ in expressions $g_1, \dots, g_n$ for its corresponding $I(g)$ (written $g_i(I)$), we obtain a classical fully specified Boolean network which we denote $\mathcal{E}(I) = \{g_1(I), \dots, g_n(I)\}$. Such a network is also called an *instance* of a partiallly specified Boolean network. We use $\mathbb{I}(\mathcal{E})$ to denote all interpretations of a given $\mathcal{E}$ and $\mathcal{I}$ is used to denote some set of selected interpretations.

All previously introduced concepts for fully specified networks can be also considered in the context of partially specified BNs. The static concepts, that are related to the state-space only, such as subspace, character, trait, and phenotypes are independent of exact BN dynamics, and we do not need a specific BN instance to consider them. On the other hand, dynamic concepts, where exact transitions between states need to be known, such as STG, attractors, or the relationship between phenotypes and attractors, need to be considered within a context of a PSBN instance $\mathcal{E}(I)$.

For example, notice the extended version of BN from Fig 2 which we show in Fig 3. We consider an external stimulus of `FGFR3`, which dictates its value (even though in this example it creates a trivial variable, which could be just a parameter on its own). Moreover, we might not be sure whether `ERK` actually has some influence on `FRS2`, hence we mark this regulation as a dashed line denoting that in some intepretations this regulation might be non-essential. We can model these partial specifications by including two zero-arity uninterpreted function symbols `FGFR3_stimulus` and `f_FRS2_by_ERK`. If we substitute `f_FRS2_by_ERK` by a value (0 or 1) the resulting fully specified function of `FRS2` is either `FGFR3` $\wedge$ $\neg$`ERK` or `FGFR3`. The full STG of all four viable BN instances can be observed in Subfigure 3c. Note, that since all uninterpreted function symbols of the network are zero-arity, they can be seen as Boolean constants. As a shorthand, we can consider an interpretation I to be a simple Boolean vector of ordered values for

these constants.

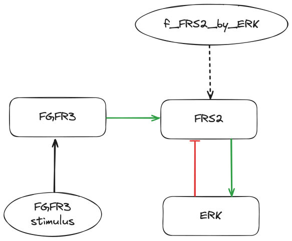

$$\mathbb{G} = \{\texttt{f\_FGFR3\_stimulus}^0, \texttt{f\_FRS2\_by\_ERK}^0\}$$
$$\texttt{FGFR3} = \texttt{FGFR3\_stimulus}()$$
$$\texttt{FRS2} = \texttt{FGFR3} \wedge (\neg\texttt{ERK} \vee \texttt{f\_FRS2\_by\_ERK}())$$
$$\texttt{ERK} = \texttt{FRS2}$$

(a) Regulatory network (b) Partially specified Boolean network

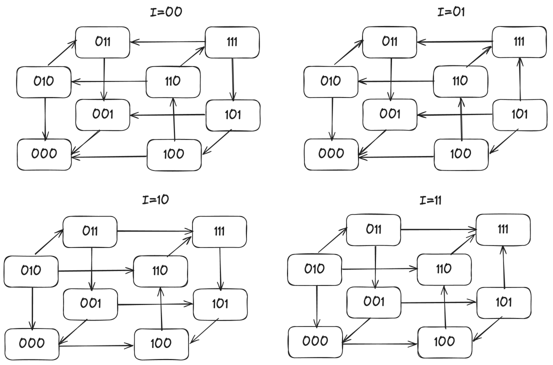

(c) PSBN instances state transition graphs

Figure 3: **An example of a partially specified Boolean network and the related concepts.** In Subfig. a an extended version of the RN from 1 is displayed. It is extended with two unary function symbols (in circles), which are having constant, but unknown value in the model. Since it is possible, that influence of one of the functional symbol might not be essential, this relationship is marked by a dashed arrow. Then, in Subfig. b we can see the corresponding PSBN. Finally, we depict the STGs of all four possible instances of the PSBN in Subfig. c.

1.5 Phenotype control of partially specified Boolean networks

Let us now focus on the topic of the phenotype control for Boolean networks: we assume a set of phenotype states and a desired relationship of the network towards the particular phenotype (stabilisation, oscillation, avoidance). The goal of phenotype control is to *perturb* the network such that all its attractors have the desired relationship to this phenotype. The perturbations on the network are typically introduced by (permanently) fixing some of the network variables

to a specific value.

As before, for clarity, we first introduce concepts for BN instances and then expand these to PSBN. First, to control the behavior of a BN, we use the notion of a *variable perturbation*.

1.5.1 Variable perturbation

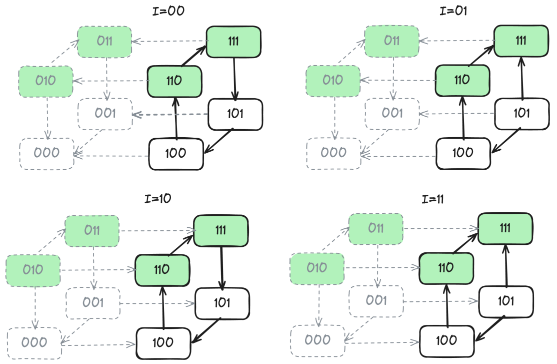

Figure 4: **An example of a perturbed state transition graph of a PSBN.** The figure shows the perturbed state transition graphs of four perturbed interpretations of the PSBN from Fig 3. The states and transitions that are disabled by the perturbation are marked with grey dashed lines, while the states and transitions which are respecting the perturbation and thus can be used are lined in bold. The target phenotype states ($\Phi = \star 1 \star$) are highlighted in green. The perturbation $Q = 1\star\star$ controls the network towards the phenotype for instances I_{01} and I_{11}, but not for I_{00} and I_{10}.

A *variable perturbation* is a vector $Q \in \mathbb{B}^n_\star$. For every $Q_i = \star$, we say that the i-th variable is *unperturbed*, whereas for $Q_i = 0$ or $Q_i = 1$, we say that the variable is *perturbed* to either 0 or 1.

We write *fixed*(Q) and *free*(Q) to denote the subset of perturbed and unperturbed variables, respectively. The *size* of Q is the size of the *fixed*(Q) set. Furthermore, a perturbation can again be seen as a *subspace* of $\mathbb{B}^n$. We call such subspace as *compliant* with Q.

For a particular Boolean network $\mathcal{F}$ and Q, we consider a *perturbed* state-transition graph $\mathrm{STG}[Q](\mathcal{F})$, which is a sub-graph of $\mathrm{STG}(\mathcal{F})$ *induced* by the states compliant with Q. Intuitively, the *fixed*(Q) variables are restricted to their perturbed values, while *free*(Q) variables are left to evolve without modification.

We say that a permanent variable perturbation $Q \in \mathbb{B}^n_\star$ *controls* Boolean network $\mathcal{F}$ towards stabilisation of phenotype Φ iff $\forall A \in \mathbb{A}(\mathrm{STG}[Q](\mathcal{F})). A \subseteq \Phi$ (all attractors of a BN stabilize in Φ). Similarly, we can also define control towards a different relationship of the network with the phenotype (avoidance, oscillation). What is also interesting, is that $\mathbb{A}(\mathrm{STG}[Q](\mathcal{F}))$ is not necessarily a subset of $\mathbb{A}(\mathrm{STG}(\mathcal{F}))$. A permanent perturbation can change existing attractors

or even introduce new ones (e.g., by elimination of outbound transitions from some states).

This concept naturally applies to interpretations of PSBNs as well: given a $\mathcal{E}$, an interpretation I and a perturbation Q, the interpretation has a perturbed $\mathrm{STG}[Q](\mathcal{E}(I))$. As such, a perturbation Q controls $\mathcal{E}$ under the interpretation I if it ensures $\mathbb{A}(\mathrm{STG}[Q](\mathcal{E}(I))) \subseteq \Phi$. An example of a perturbed STG can be seen in Fig. 4.

1.5.2 Problem definition

Assume a partially specified network $\mathcal{E}$, a phenotype $\Phi \subseteq \mathbb{B}^n$, a desired relationship $R \in \{$*stabilisation, oscillation, avoidance*$\}$ between network and phenotype, and a set of admissible perturbations $\mathbb{Q} \subseteq \mathbb{B}^n_\star$. The goal of the *complete* phenotype control is to compute all pairs $(I, Q) \in \mathbb{I}(\mathcal{E}) \times \mathbb{Q}$ such that Q controls $\mathcal{E}(I)$ towards the desired phenotype relationship R with Φ.

Intuitively, the result of PSBN phenotype control is a set of all combinations of interpretations and perturbations for which the perturbation necessarily stabilizes the associated network interpretation in attractors that have the desired relationship with the given phenotype. The set $\mathbb{Q}$ is in practice used to restrict the problem setting to perturbations of a certain size or otherwise biologically feasible perturbations.

1.5.3 Perturbation size and robustness

In practice, it is often unnecessary to compute the complete set of interpretation-perturbation pairs. The goal is instead to find the smallest functioning perturbation [31, 32]. However, in partially specified networks, such minimal perturbation typically only works for a small subset of interpretations, making it potentially unreliable in practice. To describe such a property, we consider a *robustness* metric of the phenotype control:

Assuming some partially specified network $\mathcal{E}$ and a perturbation $Q \in \mathbb{Q}$, let $\mathcal{I}_Q \subseteq \mathbb{I}$ be a set of interpretations, such that:

$$\mathcal{I}_Q = \{I \in \mathbb{I}(\mathcal{E}) \mid Q \text{ controls } \mathcal{E}(\mathcal{I})\}$$

The *robustness* ρ of a perturbation Q is then defined as:

$$\rho(Q) = \frac{|\mathcal{I}_Q|}{|\mathbb{I}(\mathcal{E})|}$$

The *robustness* ρ of a perturbation Q is then defined as a ratio of PBN interpretations for which the perturbation achieves the goal. It can also be understood as a chance that a randomly selected interpretation is controlled by the perturbation Q. Given a robustness threshold $r \in (0, 1]$, the goal of *robust* phenotype control is to compute the smallest perturbation (or perturbations) Q s.t. $\rho(Q) \geq r$.

Ideally, a suitable perturbation Q with $\rho(Q) = 1.0$ (100%) achieves control for all interpretations of $\mathcal{E}$. However, if no such perturbation exists, the parameter r can be tuned to achieve a trade-off between perturbation size and robustness.

For example, consider the network from Figure 3, a phenotype $\Phi = \star 1 \star$ and a perturbation $Q = \star\star 0$. Assume, that our goal is to stabilize in the given phenotype. Fig. 4 depicts the perturbed STGs of all possible interpretations of the network. We can easily see, that only two out of four BN instances are controlled towards the phenotype by the perturbation Q. Therefore, we can say, that Q has a robustness of 0.5 (50%).

2 Methods

In this section, we describe methods used to obtain the presented results. First, in Subsection 2.1, we describe how the phenotype control of partially specified Boolean networks is typically used in practice to design therapies and how we use it to refine PSBN models. Then, in Subsection 2.2 we zoom in on the algorithms used to solve the phenotype control problem.

2.1 Control Workflow for Therapy and Model Refinement

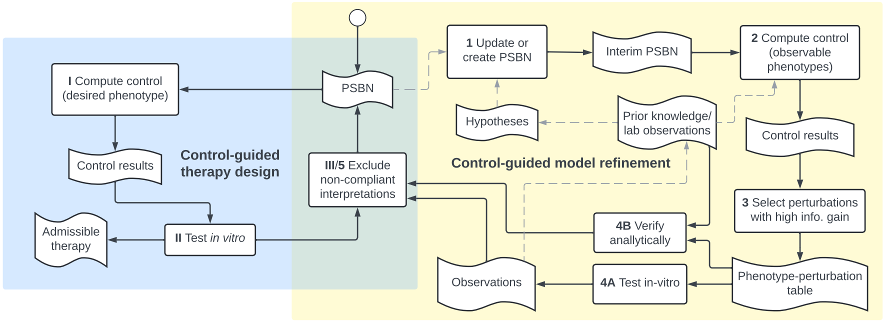

Figure 5: **Workflows of control framework.** The diagram in the picture depicts two workflows of how the phenotype control can be used to achieve two different goals – to find a therapy or to refine a PSBN model. The workflows can be combined or achieve each other's goals as a side effect (e.g., we might refine model after refuting that the obtained therapy works). The rectangle boxes represent the steps of the workflow, while the tape shaped boxes represent the artifacts obtained from the conducted steps.

The phenotype control of BNs is traditionally used as a tool to obtain therapy designs for complex diseases. The corresponding workflow is illustrated in Fig. 5 (left). The workflow starts by computing perturbations that ensure a

desired non-pathological phenotype (I). These perturbations are then validated *in vitro* by knocking out or over-expressing genes or proteins corresponding to the targeted variables (II). If the designed control strategy fails to achieve the desired phenotype experimentally, the PSBN framework can be used to exclude model instances that do not reproduce the expected behavior (III). Moreover, the relevant experimental results can inform the formulation of new hypotheses that further elucidate the system's behavior.

The impact of perturbation-based analysis on potential exclusion of non-compliant models forms a basis of our novel workflow that leverages permanent phenotype control to refine PSBN models. The general aim is to enhance existing models of biological processes through control, especially when the knowledge of mechanistic details is incomplete.

To that end, we introduce the *control-guided model refinement workflow*, schematically shown in Fig. 5 (right).

At the beginning, we assume that an abstract interim PSBN model is constructed. This model can be obtained either by confronting an existing model with new hypotheses and data (e.g., using the BN sketches framework [6]), or alternatively, by constructing a new PSBN model based on prior knowledge and known hypotheses (1). The resulting interim models typically exhibit high entropy and may not conform to the expected behavior. At this stage, the control framework is employed for computing perturbations for all observable phenotypes (2).

Next, admissible perturbations are selected—those yielding significant information gain to partition PSBN model instances into groups that exhibit distinct long-term behavior patterns (3). The selected perturbations can then be tested *in vitro* (4A) or analytically verified against prior knowledge to ensure the model behaves as expected (4B). Finally, model instances that do not conform to the expected behavior are excluded (5). The workflow can be iterated as needed.

2.1.1 Control-driven therapy design

Control-guided *therapy design* where the goal is to find a suitable treatment which achieves a desired (healthy) system behavior is a well-studied topic [7, 21, 25, 29, 36]. This objective can be directly translated to the phenotype control problem where the system behavior is described via PSBN and the target behavior via a phenotype. The output of the control problem is a set of perturbations which can be implemented back to the real-world, for example as gene knockouts and over-expressions.

In an ideal case, the artifact obtained from this workflow is a therapy design that can be proven to work in in-vitro (or in-vivo) experiments. Usually, the preferred perturbations are the ones which are easiest to implement in practice (i.e., with the smallest perturbations or the ones which are the most biologically feasible) [31]. The second criterion to consider is an expected chance of the therapy to work. This aspect is estimated by the model via a robustness metric of the perturbation [9].

Nonetheless, since the model is imprecise to some extent, the designed therapy might not work in practice. In such case the observations from the experiment can be used to refine PSBN model. Usually, we can simply refute the interpretations which do not comply with obtained observation. For example, if we expected a perturbation `FGFR3`=1 to stabilize the network in a phenotype `ERK`=1 for 60% of interpretations, but the real-world experiment do not stabilize this phenotype under `FGFR3` over-expression, we can conclude, that these 60% of interpretations are not representing the studied system correctly, and thus we can discard them from the considered set of interpretations. This would complete the workflow cycle depicted on the left side of the diagram in Fig. 5 and we can attempt to recompute the therapy design with the remaining interpretations.

2.1.2 Control-guided model refinement

In more complex cases, it is also possible, that the treatment is not replicated in vitro even for a perturbation with 100% robustness. Therefore, we might need to rework the model significantly, e.g., by introducing new model parts (regulations and variables) or by changing the existing components. Typically, these model adjustments significantly expand unknown parts of the model, since they are based on hypotheses and uncertainty. Then, a typical next step is verification of the hypotheses we posed as well as efforts to lower the introduced uncertainty.

As shown on the right side of the diagram in Fig. 5, we use phenotype control to guide an experimental design of knockout or over-expression experiments for the model refinement [33]. As opposed to the therapy design, in this case, we typically focus on perturbations which are the most informative, i.e., the ones which will help us to distinguish between the interpretations the most. More specifically, a perturbation which works for exactly same interpretations is not very informative, since we expect, that it works for all interpretations and thus would not help us lower the model uncertainty (number of interpretations).

For example, let us suppose, that we consider some PSBN $\mathcal{E}$ *similar* to the one from example in Fig. 3 (i.e., a PSBN with multiple variables and functional symbols). The PSBN has 2820 interpretations. and that we compute stabilisation in prolifertion (P) phenotype. The result of the control could be perturbations Q_A, Q_B, Q_C, while the union of the interpretations for which they work is exactly $\mathbb{I}(\mathcal{E})$. The cardinality and intersection of the interpretations they cover are shown in Figure 6a.

We can notice, that despite the perturbations Q_A and Q_C are working for exactly the same amount of interpretations, they might control very different sets of particular interpretations. If there would be multiple perturbations which work for exactly same interpretations as the perturbation Q_A, it is not expected to be informative to use them all in the real-world experiments. Finally, we can see, that the perturbation Q_B is also covering distinct sets of perturbations, although their size is much smaller.

If we were in the situation, when we would need to select only two perturbations for the real-world experiments (e.g, due to the high experiment price) we

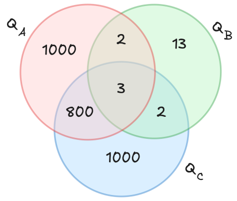

(a) Interpretation sets controlled by perturb. Q_A, Q_B, Q_C.

Q_A	Q_B	$\lvert \mathcal{I} \rvert$
P	P	5
P	A	1800
A	P	15
GA	A	1000

(b) Phenotype-perturb. table for selection of Q_A and Q_B.

Q_A	Q_C	$\lvert \mathcal{I} \rvert$
P	P	803
P	A	1002
A	P	1002
A	A	13

(c) Phenotype-perturb. table for selection of Q_A and Q_C.

Figure 6: **An example of interpretations partitioning of working perturbations.** Subfigure a shows sets of interpretations and their intersections for some three distinct perturbations Q_A, Q_B, Q_C. Then, the Subfigures b and c show phenotype-perturbation matrices if perturbations Q_A and Q_B are selected or Q_A and Q_C respectively. Each row represents a set of interpretations. The values in the table cells contain a phenotype in which model stabilizes under the given perturbation one of proliferation (P), apoptosis (A) or growth arrest (GA). The last column contains cardinality of the relevant interpretation set.

can consider the reduced interpretation sets in *perturbation-phenotype tables* depicted in Figures 6b and 6c. The rows of these tables represent non-overlapping interpretation *classes* with distinct *behavior patterns*, while column values show differing phenotype outcomes of these interpretations under the selected perturbations.

When deciding which two perturbations out of three should be kept, we would be probably inclined to select Q_A and Q_C since in the worst (and most probable) case we would reduce the model uncertainty to a third of the original interpretation cardinality. On the other hand, if we would select Q_A and Q_B, there would be a chance, that we would reduce the model uncertainty to a very small fraction of the original one, but the expected chances of this are very low. Nonetheless, the in ideal scenario we would be able to try all three perturbations in the real-world experiments, or we would prefer the perturbations which are easier to implement.

Notice, that in phenotype-perturbation matrix we may not just represent whether the perturbation works or not, but also which phenotype in particular it exhibits. This is particularly useful for the model refinement, since we can then distinguish behavior classes depending on the exhibited phenotype.

Therefore, this table can be then directly used for design of knock-out and over-expression experiments in the real world. Since rows of a table always contain a distinct combination of phenotypes, we expect to be able to match the results from the phenotypes observed in the real-world to one particular row of the phenotype-perturbation table and thus significantly reduce the model uncertainty.

2.1.3 Analytical approach to model refinement

Performing the designed experiments in vitro is an ideal approach to refine the model. However, the experiments are usually costly and might not always be possible to implement. In such cases, we can still observe which model mechanics allow or prevent a perturbation working for some interpretations. For this, purpose, we can infer *features* of an interpretation sets split obtained in the previous step.

	**Perturbations**		**Features**	
$\mid \mathcal{I} \mid$	Q_A	Q_C	$\exists I \in \mathcal{I}.\ \texttt{ERK} \to \texttt{FRS2}$	$\forall I \in \mathcal{I}.\texttt{FGFR3} = 0$
803	P	P	1	0
1002	P	A	0	1
1002	A	P	0	0
13	A	A	0	1

Table 2: **Example of phenotype-perturbation tables with extracted features.** The table shows the binarized phenotype-perturbation table for the perturbations Q_A and Q_C from Fig. 6. The table is extended with features extracted from the interpretation sets.

For example, consider Table 2, where we have selected two perturbations from the previous example in Fig. 6. In the table, we assume that we have selected perturbations Q_A and Q_C. We might be interested in finding out *why* a particular perturbation works for some interpretations and not for the others. For this purpose, we infer the features of the interpretation sets. The vector of phenotypes for perturbations can thus be considered as a *label*, and we can observe their *mutual information gain* metric to measure impact of a feature on the [20]. Note, that since question we are interested in is whether a perturbation works or not, thus it is more advantageous to consider binary labels (yes/no) for the labels.

Given a set of interpretations $\mathcal{I}$, the features we consider are following:

- A regulation is essential in all interpretations $\forall I \in \mathcal{I}.\ \texttt{GRB2} \to \texttt{FGFR3}$ is observable in $\mathcal{E}(I)$.
- A regulation is essential in any interpretation $\exists I \in \mathcal{I}.\ \texttt{GRB2} \to \texttt{FGFR3}$ is observable in $\mathcal{E}(I)$.
- A presence of a particular function instance in all of the given interpretations ($\forall I \in \mathcal{I}.\texttt{FGFR3} = \texttt{FGFR3\_stimulus} \land \neg\texttt{GRB2}$ in $\mathcal{E}(I)$). This way we learn, that a particular function is necessary for the phenotype control to work.
- A presence of a particular function instance in any of the given interpretations ($\exists I \in \mathcal{I}.\ \texttt{FGFR3} = \texttt{FGFR3\_stimulus} \land \neg\texttt{GRB2}$ in $\mathcal{E}(I)$). This way we might find, that a particular function is preventing a perturbation from working.

Note, that the features can be combined in various ways as well as there might be other features to consider (e.g. monotonity of regulations, or particular values of the function symbols, etc.). A rule of the thumb is to refine as many potentially relevant features as possible. In our example, we can notice, that perturbation Q_C avoids proliferation exactly if an interpretation has update function `FGFR3`=0. This could be a surprising observation for the modeler since maybe they did not expect `FGFR3` function to be so trivial, nor Q_C to have such effect on proliferation. If such is the case, approximately a third of the interpretations can be refuted using the analytical approach only.

In more complex cases, it becomes complicated to assess which single feature can completely predict the observed phenotypes. To quantify an extent to which the phenotype is dependent on a given feature we use the mutual information gain metric. Therefore, we consider a single vector of features at a time, and a single vector of phenotype labels yielded by a perturbation, in order to select features best describing each perturbation, to analytically evaluate mechanics of a given perturbation. Since there might be different cardinality for possible phenotype outcomes (i.e. models and perturbations might achieve different number of varying phenotypes), we use normalization for the mutual information gain [35].

To summarize, the workflow in Fig. 5 can be used to design a therapy, or guide the model refinement, or both. The cycle can be repeated any amount of times while having both of these goals in mind. The main difference between the two workflows is the selection of perturbations for the real-world experiments. In the therapy design, we are interested in the perturbations which are the most likely to work, while in the model refinement, we are interested in the perturbations which are the most informative.

2.2 Algorithms

Partially specified networks contain parameter explosion on top of the space explosion, which makes them hard to analyze exhaustively. In the context of control, this problem is further complicated by the exponential number of possible perturbations. To mitigate this, we employ symbolic state space exploration which allows us to analyze the full set of possible perturbed STGs of all the PSBN interpretations within a single algorithm pass. This technique exploits similarities between the STGs of PSBN network instances (and perturbations) to significantly speed up the exploration process compared to the naive enumeration [3, 8, 5].

2.2.1 Symbolic computation model

As we mentioned before, any partially specified Boolean network can be normalized into a special kind of PSBN which contains only nullary uninterpreted functions. This normalization allows us to represent a set of all possible interpretations as a vector of Boolean values $\mathbb{I}(\mathcal{E}) \equiv \mathbb{B}^m$.

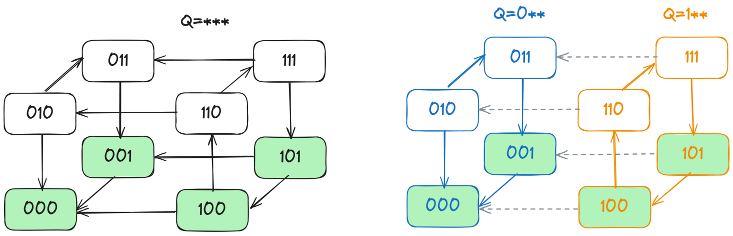

Figure 7: **An example of a perturbed STG encoding.** On the left, we can see the original STG of a BN and the target phenotype is depicted in green. On the right, we can see the version of the graph, where the first variable is perturbed. We can observe the stabilisation in phenotype ($\Phi = \star 0 \star$) in this STG and see, that for the blue part, where $Q = 0\star\star$, the network always eventually stabilizes in the phenotype and thus the perturbation is controlling while in the orange part, where $Q = 1\star\star$, the network never stabilizes in the phenotype, it will only oscillate through it ad infinitum.

A binary decision diagram (BDD) [10] is a directed acyclic graph that represents a Boolean function. A BDD-encoded function $f : \mathbb{B}^k \to \mathbb{B}$ can be used to encode a set (or relation) of Boolean vectors $X \subseteq \mathbb{B}^k$, such that $f(x) = 1 \Leftrightarrow x \in X$. Since both network states ($\mathbb{B}^n$) and interpretations ($\mathbb{B}^m$ after normalization) correspond to Boolean vectors, such sets have a direct BDD representation.

For sets of perturbations, a naive approach requires two Boolean variables per component, since their domain is $\mathbb{B}_\star$ instead of $\mathbb{B}$. However, in [9, 5], we introduced an alternative encoding suitable for representing a perturbed state-transition system that requires only one variable. This significantly improves the efficiency of the encoding, since fewer symbolic variables typically result in smaller BDDs.

The main idea behind the encoding is, that the perturbation disables a set of transitions in the STG. But we can still learn the value of the perturbed variable from the set of states themselves since the value of the perturbed value never changes. Therefore, rather, than encoding the perturbation by two variables representing the exact perturbation value, we introduce a single symbolic variable that represents that variable is perturbed. The value of the perturbed network variable can be then learned from the set of states itself. An example of such a representation can be seen in Fig. 7.

As a result, the complete encoding of a network is following:

- $\mathbb{U} \equiv \mathbb{B}^n$ encodes network states (vertices of STG($\mathbb{PSBN}$));
- $\mathbb{I} \equiv \mathbb{B}^m$ encodes network interpretations ($\mathbb{I}(\mathcal{E})$ and m is $\mid \mathbb{G} \mid$);
- $\mathbb{L} \equiv \mathbb{B}^n$ encodes sets of perturbed variables (without the actual perturbation value).

Since the method we use is only an extension of the methods introduced in the previous works [9, 5], we do not go into the details of the symbolic algorithms here. Instead, we will focus on the extension, which allows us to compute phenotype control of various relationships between the network and the phenotype (avoidance, oscillation) apart from standard stabilisation in the phenotype. We present the algorithm extension on a standard BN. The extension to PSBN is done in the same fashion as in [9, 5]. The general idea is, that the combination of $\mathbb{I} \times \mathbb{L}$ is used to symbolically represent for which interpretations and perturbations is the edge is admissible. Therefore, the space structures (such as trap sets or backward reachability) are computed for all possible combinations of interpretations and perturbations at the same time. After that, the result we obtain is a combination of interpretations and perturbations with their states (representing the actual perturbation value), which controls the network toward the desired phenotype relationship. The details of this decoding can be found in [5].

2.2.2 Supporting graph algorithms

We use two essential operations to explore the state transition graph of the BNs - function Pre to obtain all predecessors of a set of states and Post to obtain all successors respectively:

$$\textsc{Pre}(X \subseteq \mathbb{U}) = \{s \mid \exists t \in X.\ \exists i \in [1, n].\ t = s[i \mapsto f_i(s)] \wedge s \neq t\}$$
$$\textsc{Post}(X \subseteq \mathbb{U}) = \{t \mid \exists s \in X.\ \exists i \in [1, n].\ t = s[i \mapsto f_i(t)] \wedge s \neq t\}$$

Algorithm 1: Supporting graph algorithms used for phenotype control.

Fn $\textsc{Fwd}(X \subseteq \mathbb{U})$
  repeat $X \leftarrow X \cup \textsc{Post}(X)$ **until** X **reaches fixpoint**;
  return X;

Fn $\textsc{Bwd}(X \subseteq \mathbb{U})$
  repeat $X \leftarrow X \cup \textsc{Pre}(X)$ **until** X **reaches fixpoint**;
  return X;

Fn $\textsc{Trap}(X \subseteq \mathbb{U})$
  `/* Eliminate elements that escape from` X `in one step. */`
  repeat $X \leftarrow X \setminus \textsc{Pre}(\textsc{Post}(X) \setminus X)$ **until** X **reaches fixpoint**;
  return X;

The three functions from Algorithm 1 are the essential building blocks for the supporting fixed-point-based graph algorithms used for phenotype control. The first algorithm Fwd computes a set of vertices which are reachable from

the given set of vertices. Similarly, Bwd computes a complementary operation to Fwd since it computes a set of states from which the given set of vertices can be reached. Finally, algorithm Trap computes the maximal trap set of the given vertices. In our actual implementation, for the steps reaching a fixed point we employ a more efficient method to compute the maximal trap set from [4].

2.2.3 Phenotype control algorithms

Algorithm 2: Phenotype verification algorithms

```
Fn IsPhenotypeControlAux(Φ ⊆ 𝕌, osc_allowed ∈ 𝔹, init_states ⊆ 𝕌)
    phenotype_space ← if osc_allowed then Bwd(Φ) else Φ;
    phenotype_trap ← Trap(phenotype_space);
    reachable ← Fwd(init_states);
    non_phenotype ← reachable \ phenotype_trap;
    non_phenotype_trap ← Trap(non_phenotype);
    return if non_phenotype_trap = ∅ then true else false;
Fn IsPhenotypeControl(Φ ⊆ 𝕌, phen_type ∈ {stabilize, avoid, oscillate}, init_states ⊆ 𝕌)
    if phen_type = stabilize then
        return IsPhenotypeControlAux(Φ, false, init_states);
    else if phen_type = avoid then
        Φ' ← 𝕌 \ Φ;
        return PhenotypeControlAux(Φ', false, init_states);
    else if phen_type = oscillate then
        Φ' ← 𝕌 \ Φ;
        in_phen ← PhenotypeControlAux(Φ, true, init_states);
        out_phen ← PhenotypeControlAux(Φ', true, init_states);
        return in_phen ∧ out_phen;
```

Since we encode all network instances and perturbation variants into a single graph, our algorithm aims to verify whether the given network is fulfilling the control objective (i.e. the whole network has the given relationship toward the phenotype) or not. The main method for that is shown in Algorithm 2.

Even though there are three types of relationships between a network and a phenotype, the core of our algorithm IsPhenotypeControlAux relies on two different relationships – we see the oscillation as either allowed or forbidden.

In the case of a non-oscillatory phenotype, we first find the maximal trap set in the phenotype space itself. Thus, we obtain a set of states that contain all attractors stabilizing in the phenotype. We do not need to compute the specific attractors, since it is not necessary for the phenotype control and such a specific attractor refinement might be costly for big networks. After that, we verify, whether remaining reachable space contains any trap set (which would surely

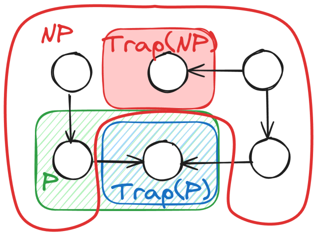

(a) BN not stabilizing in phenotype Φ due to the non-phenotype attractor.

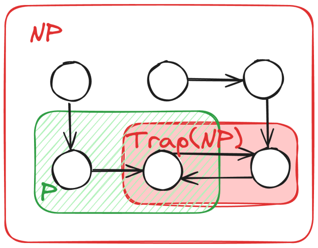

(b) BN not stabilizing in phenotype Φ due to the oscillating phenotype attractor.

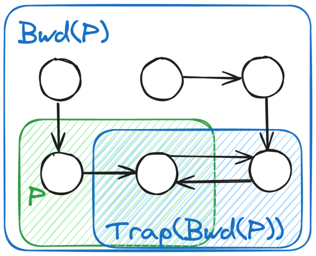

(c) BN exhibiting oscillating phenotype Φ – computation of Φ.

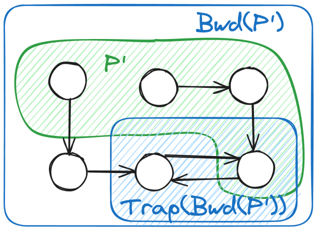

(d) BN exhibiting oscillating phenotype Φ – computation of $\Phi' = \mathbb{U} \setminus \Phi$.

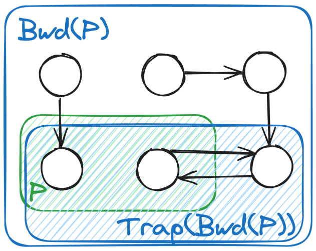

(e) BN not exhibiting oscillating phenotype Φ – computation of Φ.

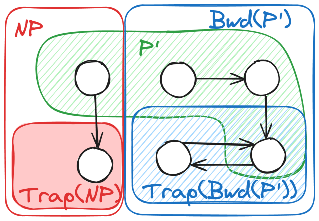

(f) BN not exhibiting oscillating phenotype Φ – computation of $\Phi' = \mathbb{U} \setminus \Phi$.

Figure 8: **Examples of algorithm 2 progressions.** Subfigures a and b show situations where BN does not stabilize in the given phenotype considering not allowed oscillation. Subfigures c and d show the computation of the oscillating phenotype Φ and its complement Φ' for the BN exhibiting the oscillating phenotype. Subfigures e and f show the same computation for the BN not exhibiting the phenotype.

contain at least one attractor). If the obtained trap set is empty, that means there are no other attractors than the ones which stabilize in the phenotype, and thus we can say, that the whole network stabilizes the phenotype. The example of an opposite situation can be seen in Fig. 8a. Also, as it can be seen from Fig. 8b, the attractors only oscillating trough Φ will be also detected as trap sets negating the phenotype, since they will not be included in $\textsc{Trap}(\Phi)$.

The function IsPhenotypeControlAux can be used as a stand-alone function when either just phenotype-stabilizing or both phenotype-stabilizing and oscillating networks are desired. Also, the phenotype avoidance algorithm is rather trivial, since it is sufficient to reverse the phenotype space as $\Phi' = \mathbb{U} \setminus \Phi$ and compute the control without allowing oscillation. However, the method IsPhenotypeControlAux on its own cannot guarantee that all attractors in the network are indeed oscillatory towards Φ.

That is why we also introduce a wrapper function IsPhenotypeControl which exactly supports the defined network-phenotype relationships. The algorithm for phenotype stabilisation and avoidance is rather trivial. For oscillatory phenotype also a rather straightforward approach is used – we verify whether the network doesn't avoid both Φ and $\Phi' = \mathbb{U} \setminus \Phi$ when the oscillation is allowed. These two steps of computation on a network that oscillates through a phenotype are illustrated in Fig. 8c and Fig. 8d. On the other hand, Figures 8e and 8f show a scenario, where the network is not oscillating because of the presence of an attractor stabilizing in Φ. Notice, that such cases are not possible to detect with a single run of IsPhenotypeControlAux.

In the end, the IsPhenotypeControl is done on all perturbed network instances simultaneously. To do that, we take advantage of our symbolic computational model. Since perturbed instances are expected to have similar STGs, this heuristic approach is expected to be more efficient than computing results on each of the graphs distinctively. The perturbed instances which do not satisfy phenotype control objectives are then discarded. As a result, we obtain a "semi-product", which we need to query, to decode the actual perturbation values and the final results. The details of this algorithm part can be found in [5].

3 Results

The Mitogen-Activated Protein Kinase (MAPK) signaling pathway is a critical cellular signaling pathway that transmits signals from the cell surface to the nucleus in response to various extracellular or internal stimuli, such as growth factors, cytokines, or DNA damage. This pathway regulates a wide range of cellular processes, including proliferation and apoptosis. The disruption of these regulations can lead to diseases, in particular various types of cancer.

We employ our methodology to analysis of two novel models. First, a smaller-scale abstract model projecting the current knowledge of FGFR3-ERK signalling is considered. Several reported unknown facts are represented by means of a PSBN and control-guided refinement is applied to precise the model

with respect to hypothesis suggested in literature. Second, we consider a larger-scale model [16] of general MAPK signalling affecting cell proliferation, apoptosis, and growth arrest. We first analyze the original model as presented in [16], and then we embed GFR3-ERK mechanism into the model and the unknown details of the signalling logics is further explored with the control-guided framework.

All our experiments were conducted using the AEON.py Python package [3], which also contains the implementation of the algorithms described in the previous section. The full source code containing all JuPyter notebooks used for the experiments along with the raw results are available at Zenodo doi `10.5281/zenodo.16886813`.

3.1 Isolated FGFR3 pathway model

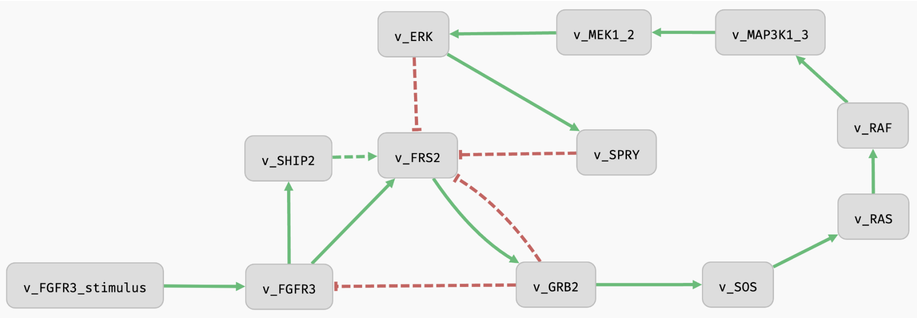

Figure 9: **An isolated FGFR3 pathway model.** The model consists of 12 variables and 16 regulatory interactions. The variables `FRS2` and `FGFR3`, are partially specified. The positive regulations are depicted in green color while negative regulations are red. The lines which are dashed represent regulations which are not required to be essential in the model interpretations. The model serves as an example for demonstrating the capabilities of our method.

Based on our previous research we consider a minimalistic model representing an abstracted form of FGFR3-MAPK signaling pathway, focusing on the interactions among FGFR3, FRS2, SHIP2 and ERK proteins [17]. The model is encoded as a PSBN consisting of 12 variables and 16 regulatory interactions. The model contains one input – `FGFR3_stimulus`, which is considered implicitly set to true to simulate constitutive activation of the pathway via suitable FGF ligands. This stimulus can be however disabled (perturbed to the value 0) with the phenotype control. The update logics of `FGFR3` and `FRS2` is partially specified reflecting the fact that the combined effect of variables regulating their activity as well as the presence of particular regulations is not well understood [13, 23].

The dynamics of the final protein component ERK closing the isolated pathway forms the (model) phenotype. In particular, we consider three protein-level

Table 3: **FGFR3 pathway model update functions.** The table shows the update functions of the FGFR3 pathway model. The variables FGFR3 and FRS2 are partially specified, while the rest of the variables are fully specified. The function symbols f, g and h are unary function symbols, while $\wedge$ and $\vee$ are binary logical operators.

Variable	Function
FGFR3	$f(\texttt{FGFR3\_stimulus}, \texttt{GRB2})$
GRB2	FRS2
FRS2	$\texttt{FGFR3} \wedge g(\texttt{ERK}, \texttt{GRB2}, \texttt{SPRY}) \vee (\texttt{FGFR3} \wedge h(\texttt{SHIP2}))$
ERK	MEK1_2
SHIP2	FGFR3
SPRY	ERK
SOS	GRB2
RAS	SOS
RAF	RAS
MAP3K1_3	RAF
MEK1_2	MAP3K1_3

phenotypes targeting ERK activity: ERK stabilisation, ERK avoidance, and ERK oscillation.

.

Due to the introduced uncertainty, the FGFR3 model has 888 possible interpretations. Due to unary function symbol design, there are only 114 semantically distinct interpretations, nonetheless, the for the computation framework we will consider all 888.

Let us we observe the non-perturbed behavior – the "natural" phenotypes of the model. After running a simple algorithm for searching the attractors supported in AEON.py, we learn that the unperturbed model stabilizes in 2 types of attractors – ERK stabilisation (556 interpretations) and ERK oscillation (332 interpretations). The ERK avoidance phenotype was not observed in the non-perturbed version of the model. This is due to the presence of the FGFR3_stimulus which we have set to true for our experiments. The stimulus transitively activates ERK via FGFR3, FRS2 and other transitive variables.

Next, we computed the phenotype control for all three considered phenotypes. The results are summarized in Table 4. Due to the small size of the model, we list only perturbations up to size one, since perturbations of bigger size did not achieve better results; not even in case of oscillation, where no perturbation has 100% robustness. Even when perturbations of all sizes were computed, no perturbation achieved better robustness towards oscillation phenotype than SHIP2=0. This might be due to the incomplete model design, which we will discuss later.

We can also notice, that perturbation of the same variable to both true and false may lead to the same phenotype (e.g. SPRY in case of oscillation). Similarly, the same perturbations can lead to multiple phenotypes (e.g. SHIP2=1).

Table 4: **Phenotype control of the isolated FGFR3 pathway model.** The table shows the perturbations that control the model towards the ERK stabilisation, avoidance and oscillation phenotypes. The symbol ∅ denotes an absence of perturbations. The robustness of each perturbation is of all interpretation of the original model is shown in the third column.

**Phenotype**	**Perturbation**	$\rho(\mathbb{I})$
ERK stabilisation	`FRS2`=1, `GRB2`=1, `MAPK3K1_3`=1, `MEK1_2`=1, `RAF`=1, `RAS`=1, `SOS`=1	1
	`FGFR3`=1	0.94
	`SPRY`=0	0.637
	∅, `SHIP2`=1, `SPRY`=1	0.626
	`SHIP2`=0	0.583
ERK avoidance	`FGFR3`=0, `FRS2`=0, `GRB2`=0, `MAPK3K1_3`=0, `MEK1_2`=1, `RAF`=0, `RAS`=0, `SOS`=0	1
	`FGFR3_stimulus`=0	0.667
	`SPRY`=1	0.017
ERK oscillation	`SHIP2`=0	0.41
	∅, `SHIP2`=1	0.374
	`SPRY`=0	0.363
	`SPRY`=1	0.357
	`FGFR3`=1	0.061
	`FGFR3_stimulus`=0	0.333

This is because in these cases the partially specified functions have a greater influence on the network dynamics, than the perturbation itself – thus, another plausible explanation is, that the network behaves the same way as without any perturbations. In case, this is not expected behavior, this can be seen as a flaw introduced in some interpretations of the model which should be therefore refuted.

To probe further all the unexpected model behavior we can use the framework of perturbation experiments (also known as knockout and over-expression experiments [33]). First, we need to observe which perturbations yield the most differing results in terms of controlled interpretations. To do this, we can depict intersections of the interpretation sets controlled by various perturbations with a robustness lesser than 100%. The results of such an analysis are displayed in Fig. 10. We use Upset plots for the visualization [24].

We can see, that `ERK` avoidance case (Subfig. 10c) gives us very straightforward answer of which perturbations should be selected, since there are only two perturbations (`FGFR3_stimulus`=0 and `SPRY`=1) covering unique sets of interpretations (the same can be seen from Table 4).

The case of `ERK` stabilisation (Subfig. 10a) is a bit more complicated, as

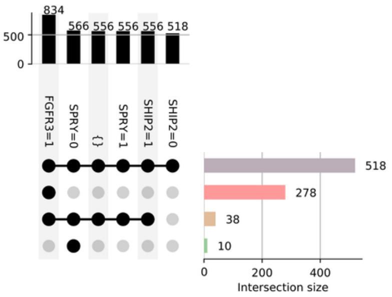

(a) ERK stabilisation.

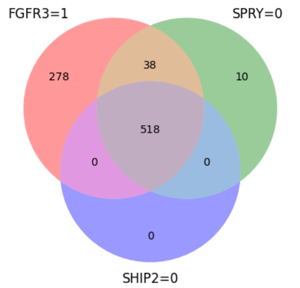

(b) ERK stabilisation - Venn diagram.

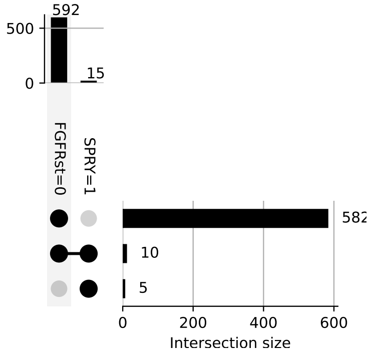

(c) ERK avoidance.

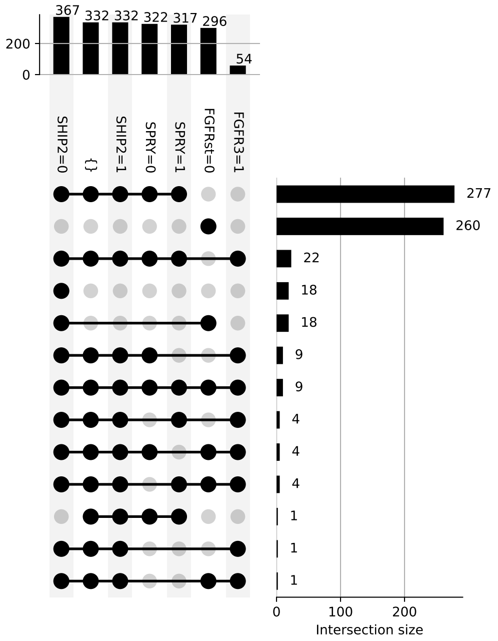

(d) ERK oscillation.

Figure 10: **Data informing selection of knockout and over-expression experiments to refine model uncertainty.** Subfigures a,c,d use UpSet plots [24] to represent intersecions between sets of interpretations controlled by particular perturbations into ERK stabilisation, avoidance and oscillation. The Subfig. b then represent intersections between controlled interpretations by perturbation candidates selected from Subfig. a.

there are multiple perturbations that could be selected. Nonetheless, we can notice, that SPRY=0 and FGFR3=1 should be selected, since they cover some unique set of interpretations. Similarly, SHIP2=0 should be selected, because it is missing some interpretations covered by all other perturbations (i.e, it is unique by interpretations which it is *not* covering). Finally, as we can see in the Subfig. 10b, these three perturbations are sufficient to cover all subset cases of the ERK stabilisation.

Last but not least, we consider phenotype control to oscillating ERK (Subfig. 10d). We can notice, that we have already selected all other perturbations to be in the perturbation experiments apart from an empty perturbation and SHIP2=1. However, these two perturbations have exactly the same behavior, and thus we can arbitrarily choose any of them – we choose an empty perturbation (since it would be expected to be easier to implement). The results of the perturbation experiments are summarized in Table 6.

Table 5: **Information gain of interpretation features.** The table shows information gain of the three best performing features (four in case of exact match) per each labelling (assessment whether a perturbation is working). The features highlighted in blue were selected to be considered in the next experiment.

Perturbation	Feature	NMIS
∅	∀SPRY→FRS2 (F_1)	0.364
	∃SPRY→FRS2 (F_2)	0.279
	∀SPRY→SHIP2	0.279
SPRY=0	∃FGFR3 = FGFR3_stimulus ∧ ¬GRB2 (F_3)	0.529
	∃GRB2→FGFR3 (F_4)	0.293
	∃FRS2 = FGFR3_stimulus	0.293
SHIP2=0	∃SPRY→FRS2 (F_2)	0.855
	∀FRS2 = FGFR3 ∧ SHIP2 (F_5)	0.735
	∃FRS2 = FGFR3 ∧ SHIP2	0.735
FGFR3_stimulus=0	∀FGFR3 = ¬GRB2 ∨ FGFR3_stimulus (F_6)	1.000
	∃FGFR3 = ¬GRB2 ∨ FGFR3_stimulus	1.000
SPRY=1	∀SPRY→FRS2 (F_1)	0.485
	∃FRS2 = FGFR3 ∧ ¬SPRY (F_7)	0.437
FGFR3=1	∃SHIP2→FRS2 (F_8)	0.562
	∀SPRY→FRS2 (F_1)	0.562
	∃SPRY→FRS2 (F_2)	0.442
	∀SHIP2→FRS2	0.442

Before diving into the results of the interpretation split and model behavior under differing perturbations we might first pose the question: "Why a perturbation works for some particular set of interpretations and not for others?" in

order to better explain the observed model behavior. To inquire that, we can look into a set of common *features* of the labelled interpretations, where *label* is an assessment which phenotype (ERK avoidance, activation, or oscillation) is manifested by a given interpretation.

Table 6: **Behavior of isolated FGFR3 pathway interpretations under perturbations.** Each row reflects interpretations sharing a particular behaviour pattern (ERK exhibiting specific phenotypes under considered perturbation experiments). The first column displays a row index while the second column indicates how many interpretations exhibit the displayed behavior pattern. The second column describes ERK phenotypes achieved under the listed knockout/over-expression experiments, in particular: no perturbation (∅), `SPRY`=0 (Q_1), `SHIP2`=0 (Q_2), `FGFR3_stimulus`=0 (Q_3), `SPRY`=1 (Q_4), and `FGFR3`=1 (Q_5). The values denote the phenotypes: `ERK` stabilisation (1), avoidance (0), or oscillation ($\rightleftarrows$). The last column represents relevant model features, i.e., the fact whether all corresponding interpretations fullfill them: $\exists$`SPRY`$\rightarrow$`FRS2` (F_1), $\forall$`SPRY`$\rightarrow$`FRS2` (F_2), $\exists$`FGFR3` = `FGFR3_stim.` $\wedge\neg$`GRB2` (F_3), `GRB2`$\rightarrow$`FGFR3` (F_4), $\forall$`FRS2` = `FGFR3` $\wedge$ `SHIP2` (F_5), $\forall$`FGFR3` = $\neg$`GRB2` $\vee$ `FGFR3_stimulus` (F_6), $\forall$`FRS2` = `FGFR3` $\wedge$ $\neg$`SPRY` (F_7), and $\forall$`SHIP2`$\rightarrow$`FRS2` (F_8). Rows highlighted in gray allow non-trivial `ERK` activity even with `FGFR3_stimulus=0`, violating our first assumption. The three green rows are the rows which satisfy both of our control-guided assumptions.

#	$\lvert\mathcal{I}\rvert$	ERK under perturbation						Features of $\mathcal{I}$							
		∅	Q_1	Q_2	Q_3	Q_4	Q_5	F_1	F_2	F_3	F_4	F_5	F_6	F_7	F_8
1	277	$\rightleftarrows$	$\rightleftarrows$	$\rightleftarrows$	0	$\rightleftarrows$	1	0	1	1	1	0	0	0	1
2	259	1	1	1	0	1	1	0	0	0	0	0	0	0	0
3	259	1	1	1	$\rightleftarrows$	1	1	0	0	0	1	0	1	0	0
4	22	$\rightleftarrows$	$\rightleftarrows$	$\rightleftarrows$	0	$\rightleftarrows$	$\rightleftarrows$	0	1	1	1	0	0	0	0
5	18	1	1	$\rightleftarrows$	0	1	1	0	1	0	0	0	0	0	1
6	18	1	1	$\rightleftarrows$	$\rightleftarrows$	1	1	0	1	0	1	0	1	0	1
7	9	$\rightleftarrows$	$\rightleftarrows$	$\rightleftarrows$	0	0	$\rightleftarrows$	1	1	1	1	0	0	1	0
8	9	$\rightleftarrows$	$\rightleftarrows$	$\rightleftarrows$	$\rightleftarrows$	$\rightleftarrows$	$\rightleftarrows$	0	1	0	1	0	1	0	0
9	4	$\rightleftarrows$	1	$\rightleftarrows$	0	$\rightleftarrows$	$\rightleftarrows$	1	1	0	0	0	0	0	0
10	4	$\rightleftarrows$	1	$\rightleftarrows$	$\rightleftarrows$	$\rightleftarrows$	$\rightleftarrows$	1	1	0	1	0	1	0	0
11	4	$\rightleftarrows$	$\rightleftarrows$	$\rightleftarrows$	$\rightleftarrows$	0	$\rightleftarrows$	1	1	0	1	0	1	0	0
12	1	$\rightleftarrows$	$\rightleftarrows$	0	0	$\rightleftarrows$	0	0	0	1	1	1	0	0	1
13	1	$\rightleftarrows$	1	$\rightleftarrows$	$\rightleftarrows$	0	$\rightleftarrows$	1	1	0	1	0	1	1	0
14	1	1	1	0	0	1	1	0	0	0	0	1	0	0	1
15	1	1	1	0	$\rightleftarrows$	1	1	0	0	0	1	1	1	0	1
16	1	$\rightleftarrows$	1	$\rightleftarrows$	0	0	$\rightleftarrows$	1	1	0	0	0	0	1	0

Having a derived set of features and labels, we can compute *normalized mutual information score* (NMIS) [20] to see which of the features are the most informative per each labelling. We use the following formulas to compute NMIS:

$$IS(X;Y) = \sum_{x,y} P(x,y) \log \frac{P(x,y)}{P(x)P(y)}$$

$$H(X) = -\sum_x P(x) \log P(x)$$

$$NMIS(X;Y) = \frac{2IS(X;Y)}{H(X)H(Y)}$$

The best performing results of this analysis are shown in Table 5. The full results are in files 01_mapk_small_fgfr3_isolated_features_info_score_*.csv of the Zenodo archive.

For each perturbation in Table 9, we select two features with the biggest NMIS, unless we obtain complete information (1.0) from a single feature. Therefore, we obtain eight distinct features in total, as can be seen in Table 5.

Next, we can analyze perturbation-phenotype table extended with most representative features in Table 6. We can also demonstrate how analytical observations may easily narrow down sensible interpretations. For example, we may notice, that the rows #2 and #14 of the table never lead to the ERK oscillation which can happen only in the absence of cyclic regulations in the partially specified network. This is also reflected by absence of any regulation features for row #2 as can be seen in detail in the full feature table 5. Even though the row #14 contains one cycle inducing regulations, it is not sufficient to expose the oscillation behavior for any interpretation, and thus we refute this interpretation as well.

Another types of interpretations which we refute are the ones where ERK oscillates under `FGFR3_stimulus`=0 (rows #3, 6, 8, 10, 11, 13, 15). The original intention of the model was, that `FGFR3_stimulus` is required for any `ERK` activation. We can also see that exactly the oscillating rows are the ones which contain the feature `FGFR3` = ¬`GRB2` ∨ `FGFR3_stimulus`. This shows, that ¬`GRB2` is sufficient to activate `FGFR3` and consequently `ERK`, what is in conflict with the intended design.

Finally, we have made a literature search to identify experimental biology results testing perturbations `SHIP2`=0 (Q_2) and `FGFR3_stimulus`=0 (Q_3), since these perturbations have most prominent efects based on the analytic observations. Studying the recent biological literature related to these signalling components have lead us to declare the following assumptions: First, based on [13], we expect `FGFR3_stimulus` to be *necessary* to achieve `ERK` stabilisation or oscillation in this isolated model. Second, based on [12], we observe that `SHIP2=0` knock-out causes down-regulation of `ERK` activity.

To put the literature-based assumptions into context, we can easily identify that only 9 equivalence classes (namely 1,2,4,5,7,9,12,14,16) adhere to our first assumption, completely eliminating `ERK` activity in response to `FGFR3_stimulus=0` (Q_3) perturbation. Out of these nine options, the second assumption allows to select rows 5, 12, and 14, as these are the only cases where the activity of `ERK` is plausibly down-regulated compared to the unperturbed network in response to `SHIP2=0` knock-out (Q_2), resulting in 20/888 candidate networks.

To disambiguate between rows 5, 12, and 14, we would have to quantify the effects observed by [12]. However, based on the fact that [12] do not report `ERK` to be completely inactive, we can safely eliminate rows 12 and 14 (where `ERK`

phenotype drops to 0). Furthermore, considering the fact that `ERK` is known to oscillate [28], we prefer row 5 as the most likely option.

3.2 Grieco MAPK

Another model we consider is the Grieco et al. version of MAPK model as presented in [16]. This model represents a bladder cancer behavior using a combination of three pathways (FGFR3, EFGR, and TGFBR). The model has four inputs (a stimulus for each pathway and a DNA damage). We consider these inputs set to a constant false with an exception to FGFR3 which set to true (as in the previous model).

The regulatory network of the model can be found in Figure 11. The exact function definitions are included in Table 7.

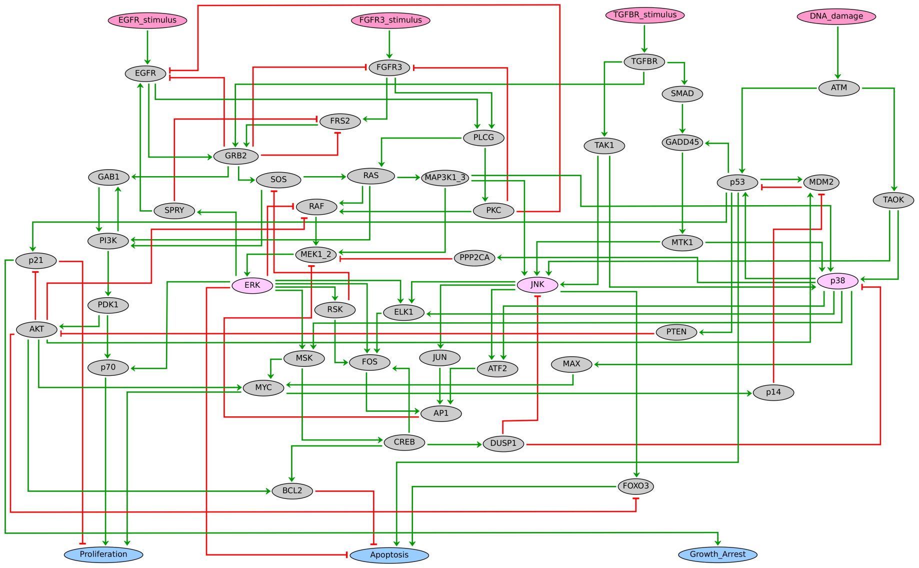

Figure 11: **Regulatory network of the MAPK signalling pathway model.** The model consists of 53 variables and 104 regulatory interactions. The model is directly taken from [16]

Moreover, the MAPK model contains three outputs (apoptosis, growth arrest, proliferation). These outputs are then used to represent the four cardinal phenotypes of the model. These phenotypes and their exact configuration is depicted in Table 8.

In the study [16], the full model version with 53 variables was presented. However, the simulations of knockout and over-expression experiments were performed on a reduced variant of the model (having only 17 variables) to observe how they impact the model phenotypes. Our novel approach allows us to consider the full model and all possible perturbations of the model at

Table 7: **Update functions of the isolated FGFR3 pathway modexl.** The table shows the update functions of the variables in the model as in [16]. The variables are ordered alphabetically. The last four variables are inputs of the model.

Variable	Function
AKT	PDK1 ∧ PTEN
AP1	JUN ∧ (ATF2 ∨ FOS)
ATF2	JNK ∨ p38
ATM	DNA_damage
Apoptosis	(FOXO3 ∧ p53) ∧ ¬(BCL2 ∨ ERK)
BCL2	CREB ∧ AKT
CREB	MSK
DUSP1	CREB
EGFR	(EGFR_stimulus ∧ ¬(PKC ∨ GRB2)) ∨ (SPRY ∧ ¬(PKC ∨ GRB2))
ELK1	(JNK ∨ ERK) ∨ p38
ERK	MEK1_2
FGFR3	FGFR3_stimulus ∧ ¬(PKC ∨ GRB2)
FOS	ERK ∧ RSK ∧ (CREB ∨ ELK1)
FOXO3	JNK ∧ ¬AKT
FRS2	FGFR3 ∧ ¬(GRB2 ∨ SPRY)
GAB1	PI3K ∨ GRB2
GADD45	p53 ∨ SMAD
GRB2	EGFR ∨ TGFBR ∨ FRS2
Growth_Arrest	p21
JNK	(TAOK ∧ MAP3K1_3) ∨ (TAK1 ∧ ¬DUSP1) ∨ (MAP3K1_3 ∧ MTK1) ∨ (TAK1 ∧ MAP3K1_3) ∨ (MAP3K1_3 ∧ ¬DUSP1) ∨ (TAK1 ∧ v_MTK1) ∨ (TAK1 ∧ TAOK) ∨ (TAOK ∧ MTK1) ∨ (MTK1 ∧ ¬DUSP1) ∨ (TAOK ∧ ¬DUSP1)
JUN	JNK
MAP3K1_3	RAS
MAX	p38
MDM2	(AKT ∧ ¬p14) ∨ (p53 ∧ ¬p14)
MEK1_2	(RAF ∧ ¬(PPP2CA ∨ AP1)) ∨ (MAP3K1_3 ∧ ¬(PPP2CA ∨ AP1))
MSK	p38 ∨ ERK
MTK1	GADD45
MYC	(MSK ∧ AKT) ∨ (MSK ∧ MAX)
PDK1	PI3K
PI3K	GAB1 ∨ (RAS ∧ SOS)
PKC	PLCG
PLCG	EGFR ∨ FGFR3
PPP2CA	p38
PTEN	p53
Proliferation	p70 ∧ MYC ∧ ¬p21
RAF	(PKC ∧ ¬(ERK ∨ AKT)) ∨ (RAS ∧ ¬(ERK ∨ AKT))
RAS	SOS ∨ PLCG
RSK	ERK
SMAD	TGFBR
SOS	GRB2 ∧ ¬RSK
SPRY	ERK
TAK1	TGFBR
TAOK	ATM
TGFBR	TGFBR_stimulus
p14	MYC
p21	p53 ∧ ¬AKT
p38	(MAP3K1_3 ∧ MTK1) ∨ (TAK1 ∧ ¬DUSP1) ∨ (TAOK ∧ MAP3K1_3) ∨ (MAP3K1_3 ∧ ¬DUSP1) ∨ (TAK1 ∧ MAP3K1_3) ∨ (TAOK ∧ ¬DUSP1) ∨ (TAK1 ∧ MTK1) ∨ (TAK1 ∧ TAOK) ∨ (MTK1 ∧ ¬DUSP1) ∨ (TAOK ∧ MTK1)
p53	(p38 ∧ ¬MDM2) ∨ (ATM ∧ ¬MDM2) ∨ (ATM ∧ p38)
p70	PDK1 ∧ ERK
DNA_damage	FALSE
EGFR_stimulus	FALSE
FGFR3_stimulus	TRUE
TGFBR_stimulus	FALSE

Table 8: **MAPK phenotypes.** The cardinal phenotypes of the Grieco MAPK model which will be used throughout the experiments. The first two columns represent the full name and abbreviation of the phenotype. The last three columns represent the model output values which are representing a given phenotype.

Phenotype	Abbreviation	Apoptosis	Growth arrest	Proliferation
Apoptosis	A	1	*	0
Growth arrest	GA	0	1	0
Proliferation	P	0	0	1
No decision	ND	0	0	0

once, which helps us to obtain more comprehensive results. Table 9 shows the exhaustive enumeration of one-size perturbations which lead to any phenotype (as in Table 8), including all observed combinations of phenotype oscillations.

We can notice, that stabilisation in all phenotypes apart from the growth arrest is possible with perturbations of size one. The unperturbed model behavior is an oscillation through all the phenotypes. We can also observe, that if the only input which we had set to true (`FGFR3_stimulus`) is disabled, the model stabilizes in a no decision phenotype.

We can also observe the influence of ERK, which was representing the phenotype in the previous model. Since its perturbation achieves a proliferation, it is indeed a strong driver of the proliferation phenotype. The other proliferation drivers were observed as a stabilizing factors of ERK in the previous reduced model, suggesting the transitive effect on proliferation. Multiple previously observed ERK stabilisation drivers are however not present in the perturbations of the full model. This can be due to the fact, that the full model contains more variables and thus the influence of ERK is more complex.

3.3 Extended FGFR3 Pathway MAPK

To demonstrate our method on a larger-scale model, we extend the fully specified model [16] that puts FGFR3-MAPK signalling into a broader context of protein interactions having the crucial impact on cell growth/division. The model has originally targeted bladder tissue cells. Our extension employs the PSBN framework allowing us to establish a partially specified model that incorporates mechanisms which are not yet understood well. In particular, based on Reactome [26] and state-of-the-art knowledge [12, 11] we compile an extended model bringing the updated FGFR3 activation mechanism to the Grieco model. This is done by embedding the isolated FGFR3-MAPK pathway studied in previous section. At the level of regulations, the embedding brings in: (1) the addition of SHIP2 affecting the FRS2 adapter phoshorylation capabilities as discussed within the isolated model, (2) addition of the direct negative feedback from ERK to FRS2 (experimentally studied mostly in the context of chondrocytes), (3) making all negative feedback regulations affecting FGFR3 and FRS2

Table 9: **MAPK perturbations.** A list of perturbations of size up to 1 which drive the MAPK model to a phenotype given in the first column. An ∅ symbol stands for no perturbation while perturbations contained in the square brackets are needed to be combined in order to achieve the perturbation goal. The last column contains a count of unique smallest perturbations achieving the given phenotype.

**Phenotype**	**Perturbations**	#
Apoptosis	ATM=1; CREB=0; DNA_damage=1; DUSP1=0; FRS2=1; GRB2=1; TAK1=1; TAOK=1; TGFBR=1; TGFBR_st.=1	10
Growth arrest	[AKT=p21=1]; [ATM=BCL2=1]; [ATM=ERK=1]; [ATM=1,FOXO3=0]; [ATM=1,JNK=0]; ...	85
No decision	FGFR3=0; FGFR3_stimulus=0; MAP3K1_3=0; MSK=0; MYC=0; PKC=1; RAS=0	7
Proliferation	ERK=1; MEK1_2=1; RAF=1	3
⇄A-GA	p21=1, p38=1, p53=1	3
⇄A-ND	[AP1=1,p21=0]; [ERK=p21=0]; [GRB2=p21=0]; [JNK=1,p21=1]; [MEK1_2=p21=0]; ...	10
⇄A-P	N/A	0
⇄GA-ND	[AP1=BCL2=1]; [AP1=1,FOXO3=0]; [AP1=1=JNK=0]; [BCL2=1,ERK=0]; [BCL2=1,GRB2=0]; ...	26
⇄GA-P	N/A	0
⇄ND-P	AKT=1; CREB=1; DUSP1=1; MDM2=1; MSK=1; PTEN=0; p14=0; p21=0; p38=0; p53=0	10
⇄A-GA-ND	AP1=1; ERK=0; GRB2=0; JNK=1; MEK1_2=0; PDK1=0; PI3K=0; PPP2CA=1; p70=0	9
⇄A-GA-P	N/A	0
⇄A-ND-P	p21=0	1
⇄GA-ND-P	BCL2=1; FOXO3=0; JNK=0	3
⇄∀	∅	1

Table 10: **Changes done to model MAPK model.** The table shows the changes done to the original model [16] in order to obtain the extended model. The first column shows the variable and a type of the change. The second column shows the original list of regulations or functions. The third column shows the new list of regulations or functions with the changes highlighted in blue font. The regulations marked with + are positives, - are negatives and ? denotes that regulation might not be present in an interpretation. Double ?? denotes that the regulation might be present in an interpretation as both positive and negative. Functions are denoted with f and g are the functions which are not specified in the model, but their instance must adhere to the listed regulations.

	Original	New
`FGFR3` reg.	`FGFR3_stim`+, `GRB2`-, `PKC`-	`FGFR3_stim`+, `GRB2`-?, `PKC`-?
`FGFR3` def.	`FGFR3_stim` $\wedge \neg$(`GRB2` $\vee$ `PKC`)	`FGFR3_stim` $\wedge$ f(`GRB2`,`PKC`)
`FRS2` reg.	`FGFR3`+, `GRB2`-, `SPRY`-	`ERK`-?, `FGFR3`+, `GRB2`-?, `SHIP2`??, `SPRY`-?
`FRS2` def.	`FGFR3` $\wedge \neg$(`GRB2` $\vee$ `SPRY`)	g(`FGFR3`,`ERK`,`GRB2`,`SHIP2`,`SPRY`)

non-essential.

At the level of logical rules, the update functions of `FGFR3` and `FRS2` are made unspecified with the only constraint that `FGFR3_stimulus` is left as a necessary precursor of `FGFR3` activation. The chosen level of abstraction reflects the fact that biochemical mechanisms specifying how the respective regulations affecting `FGFR3` and `FRS2` are combined are not currently known. Same as before, we consider the inputs to be fixed to 0, with an exception to FGFR3 stimulus. The resulting model contains 54 variables and four cardinal phenotypes as described in [16] – apoptosis (A), growth arrest (GA), no decision (ND), and proliferation (P). The exact overview of the model changes is available in the Table 10.

The goal of the analysis is to apply control-guided refinement to identify perturbations that can reduce the set of possible interpretations of the extended PSBN. We consider the hypotheses on the FGFR3 signalling mechanisms stated in previous section and the pior knowledge based on [16, 34, 30].

First, we compute control for all phenotypes including oscillations among them (step 2 of the control-guided model refinement). We restrict the results to perturbations of size up to 1. The performance results are shown in Table 11. We can see, that our method was able to compute perturbations for all cardinal phenotypes in a reasonable time. What we can also notice is that the oscillation phenotypes are more difficult to compute, which is expected since they are more complex as can be seen in the Algorithm 2 where the oscillation control requires the computation of a trap set on a bigger state space on both the phenotypes and its complement. Also, the proliferation oscillation phenotype is being computed for particularly long time. This looks like a consequence of the long computation of the proliferation avoidance phenotype. Since we are computing the trap set on an extension of this phenotype, the long computation time is expected. Since all phenotypes have the similar size of state space (with

Table 11: **Extended MAPK model performance.** The table shows the performance of the extended MAPK model. The first column shows the phenotype, the second column shows the relation to the perturbation (standard, avoid, oscillation), and the third column shows the time of the computation. The computations were performed on a computer with AMD Ryzen Threadripper 2990WX 32-Core Processor and 64GB of memory.

Φ	**relation**	**time**
GA	standard	00:02:45
P	standard	00:24:59
A	standard	00:35:59
A	avoid	00:39:26
ND	avoid	00:40:00
GA	avoid	00:41:56
ND	standard	01:13:09
GA	oscillation	01:17:13
A	oscillation	02:42:08
P	avoid	03:50:26
ND	oscillation	04:05:15
P	oscillation	13:09:52

an exception to apoptosis), we can also see, that the time of the computation is not directly related to the size of the phenotype, but rather to the complexity of the phenotype and its complement.

The actual computed control results are shown in Table 12. We can see, that perturbations can achieve all the four cardinal phenotypes as well as various oscillations among them. We can also observe the correlation between the ERK activity and the proliferation phenotype—in particular, the stabilisation of $\{\texttt{ERK} \mapsto 1\}$ is required for the proliferation phenotype while apoptosis phenotype implies avoidance of $\{\texttt{ERK} \mapsto 1\}$.

The results highlighted in blue correspond with the perturbations computed on the original model [16] (see Table 9). Moreover, the results include several perturbations that have not been observed before. Notably, there is the perturbation `PLCG`=0 which leads to apoptosis stabilisation. This perturbation is not present in the original model, however, it has been observed in [34, 30] that PLCG is a driver for cell proliferation while its inhibition can lead to apoptosis. This is a strong evidence that this perturbation is indeed valid, and we can use it for our model refinement.

Based on the discussion above, the perturbations `ERK`=1 (achieving proliferation) and `PLCG`=0 (leading to apoptosis) are strongly affecting the long-term behavior of the model. To that end, we use this knowledge to complete the first iteration of the control-guided refinement procedure.

Next, we re-compute the robustness values for the reduced set of interpretations satisfying the properties discussed in the previous paragraph used as the reference set $\mathcal{I}$ (the last column of Table 12).

Next, we select perturbations achieving partitioning to distinct behavior patterns. Due to a big number of viable perturbations with non-100% robustness,

Table 12: **Phenotype control of extended MAPK model.** The first column shows the (stable) phenotype or multiple oscillating phenotypes manifested by the model under perturbations listed in the second column. Perturbations in blue font are working in the original [16] model. Perturbations highlighted in green are the reference perturbations we used for the model refinement. The third column displays the long-term ERK activity, and the last column shows the robustness of respective perturbations. The symbol ’*’ denotes that we observe amiguous ERK behavior under the given perturbations.

Φ	**Perturbations**	ERK	ρ for $\mathbb{I}$	ρ for $\mathcal{I}$
A	ATM=1,CREB=0,DNA_dmg=1,DUSP1=0, TAK1=1,TAOK=1,TGFBR=1,TGFBR_st.=1	0	1	1
	PLCG=0	0	0.80	1
	FRS2=1,GRB2=1	0	0.64	1
	AP1=1,ERK=0,GADD45=1,JNK=1,JUN=1, MEK1_2=0,MTK1=1,PPP2CA=1,SMAD=1, p38=1,p53=1,SHIP2=1,SHIP2=0,PKC=1, ∅,SPRY,RSK=0,PKC=0,SPRY=1	0	<0.6	<0.5
GA	BCL2=1,FOXO3=0,JNK=0	0	0.61	0
	ERK=1,MEK1_2=0,p21=1	1	<0.3	0
ND	AKT=1,PTEN=0;p38=0	0	0.61	0
	MAP3K1_3=0,MSK=0,MYC=0,RAS=0	*	0.50	1
	AKT=0,FGFR3=0,GAB1=0,RSK=1,SOS=0, FGFR3_stimulus=0,p53=0,p70=0, PDK1=0,PI3K=0,PKC=1,PLCG=0,PTEN=1	*	<0.3	<0.2
P	ERK=1, MEK1_2=1	1	0.3	1
	RAF=1	1	0.19	1
	GAB1=1,GADD45=0,MAX=0,MDM2=1, MTK1=0,p14=0,p53=0,PDK1=1,PI3K=1	1	<0.1	0
A-GA	p38=1,p53=1	0	0.25	1
	p21=1	*	0.19	1
ND-P	p53=0	$\rightleftarrows$	0.55	1
	MDM2=1,p14=0,AKT=1,CREB=1,DUSP1=1, MSK=1,PTEN=0,p38=0	$\rightleftarrows$	<0.3	1
A-GA-ND	AP1=1,ERK=0,JNK=1,MEK1_2=0,PPP2CA=1	0	0.25	1
	GRB2=0	$\rightleftarrows$	0.2	0.6
	PDK1=0, PI3K=0, p70=0	$\rightleftarrows$	<0.2	1
A-ND-P	p21=0	$\rightleftarrows$	0.19	1
GA-ND-P	BCL2=1; FOXO3=0; JNK=0	$\rightleftarrows$	0.19	1
$\rightleftarrows\forall$	∅	$\rightleftarrows$	0.28	1

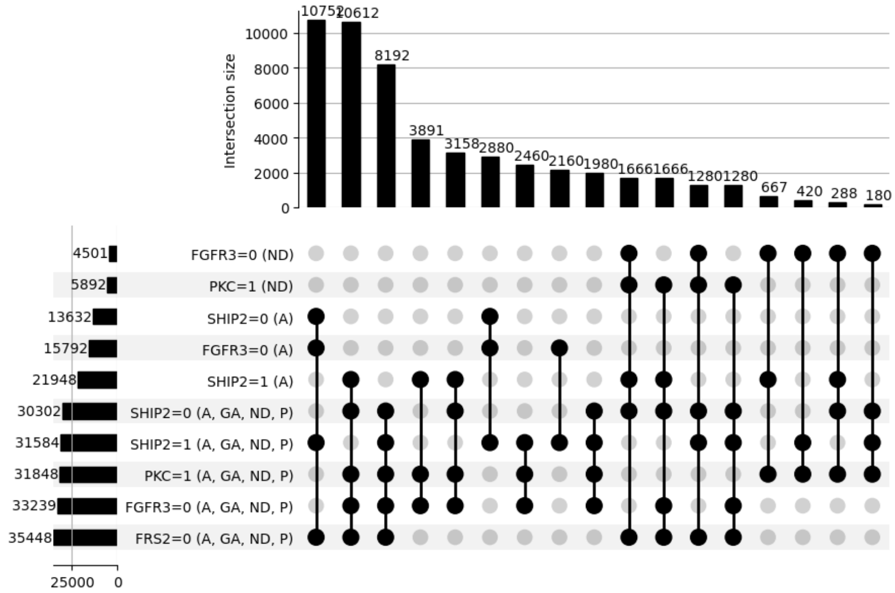

Figure 12: **Upset plot for the FGFR3 extended model.** The plot clearly shows, that each perturbation achieves a unique interpretation partitioning. Moreover, it is not possible to achieve two different phenotypes at once using the same perturbation. The perturbations are not unique, since we consider all phenotypes and perturbations at once.

we first cross-compared all combinations of perturbations and phenotypes they achieve, to obtain a set of perturbation equivalence classes. Such equivalence classes were 10 having 1-3 members. Then, we selected one perturbation from each equivalence class. To verify, that they are indeed unique, we show their intersections in Figure 12. Since we consider all phenotypes and perturbations at once, the perturbations are not unique. With upset diagram, we confirmed that all perturbations help with the interpretation classes partitioning unique and, that no perturbation achieves two different phenotypes at once what is expected for the correctness of our method.

As a result we obtain only five distinct perturbations promising for the model refinement. The perturbations are shown in Table 13. The table displays the admissible perturbations that can be considered for *in vitro* perturbation experiments design leading to potential exclusion of incomplient interpretations of the PSBN. The wet lab testing is necessary to complete the second iteration of the model refinement process, as illustrated in Figure 5.

We can see that some perturbations can produce bi- or tri-stable behaviour. However, this appears to be critical only for `SHIP2`=0 perturbation, where we might struggle to differentiate between overlapping bi-stability or tri-stability outcome. In that case we might not be able to establish a single behavior

Table 13: **Behavior of interpretations under perturbations of extended MAPK model.** The first column denotes the size of the interpretation set, while the next five columns denote the behavior of the interpretations under the selected perturbations. The | separates possible bi-stability outcome while ⇄ denotes oscillation between all model phenotypes.

$\mid \mathcal{I} \mid$	FGFR3=0	FRS2=0	PKC=1	SHIP2=0	SHIP2=1
10752	A	⇄	A	A	⇄
10612	⇄	⇄	⇄	⇄	A
8192	⇄	⇄	⇄	⇄	⇄
3848	⇄	A|ND|P	⇄	ND|P	A
3158	⇄	A|ND|P	⇄	⇄	A
2880	A	A|ND|P	A	A	⇄
2430	⇄	A|ND|P	⇄	ND|P	⇄
2160	A	A|ND|P	A	A|ND|P	⇄
1980	⇄	A|ND|P	⇄	⇄	⇄
1666	ND	⇄	ND	⇄	A
1666	⇄	⇄	ND	⇄	A
1280	ND	⇄	ND	⇄	⇄
1280	⇄	⇄	ND	⇄	⇄
624	ND	A|ND|P	⇄	ND|P	A
390	ND	A|ND|P	⇄	ND|P	⇄
288	ND	A|ND|P	⇄	⇄	A
180	ND	A|ND|P	⇄	⇄	⇄
43	ND	A|ND|P	⇄	A|ND|P	A
43	⇄	A|ND|P	⇄	A|ND|P	A
30	⇄	A|ND|P	⇄	A|ND|P	⇄
30	ND	A|ND|P	⇄	A|ND|P	⇄

class (e.g., rows #14 and #18 differ only in SHIP2=0—if relevant perturbation experiments do not exhibit (stable) apoptosis, we cannot distinguish between the two classes of interpretations).

References

[1] Réka Albert. “Boolean modeling of genetic regulatory networks”. In: *Complex Networks*. Springer, 2004, pp. 459–481.

[2] Roberto Barbuti et al. “A survey of gene regulatory networks modelling methods: From differential equations, to Boolean and qualitative bioinspired models”. In: *Journal of Membrane Computing* 2.3 (2020), pp. 207–226.

[3] Nikola Beneš et al. “AEON.py: Python library for attractor analysis in asynchronous Boolean networks”. In: *Bioinformatics* 38.21 (2022), pp. 4978–4980.

[4] Nikola Beneš et al. “BDD-based algorithm for SCC decomposition of edge-coloured graphs”. In: *Logical Methods in Computer Science* 18 (2022).

[5] Nikola Beneš et al. “Phenotype Control of Partially Specified Boolean Networks”. In: *CMSB*. Cham: Springer Nature Switzerland, 2023, pp. 18–35.

[6] Nikola Beneš et al. “Boolean Network Sketches: A Unifying Framework for Logical Model Inference”. In: *Bioinformatics* (2023).

[7] Peter Bloomingdale et al. “Boolean network modeling in systems pharmacology”. In: *Journal of pharmacokinetics and pharmacodynamics* 45 (2018), pp. 159–180.

[8] Luboš Brim et al. “Parallel one-step control of parametrised Boolean networks”. In: *Mathematics* 9.5 (2021), p. 560.

[9] Luboš Brim et al. “Temporary and permanent control of partially specified Boolean networks”. In: *Biosystems* 223 (2023), p. 104795. ISSN: 0303-2647.

[10] Randal E. Bryant. “Graph-based algorithms for Boolean function manipulation”. In: *IEEE Transactions on Computers* 35.8 (1986), pp. 677–691.

[11] B. Fafilek et al. “Expanding horizons of achondroplasia treatment: current options and future developments”. In: *Osteoarthritis and Cartilage* 30.4 (2022), pp. 535–544. ISSN: 1063-4584. DOI: `https://doi.org/10.1016/j.joca.2021.11.017`.

[12] Bohumil Fafilek et al. “The inositol phosphatase SHIP2 enables sustained ERK activation downstream of FGF receptors by recruiting Src kinases”. In: *Science signaling* 11.548 (2018), eaap8608.

[13] Silvie Foldynova-Trantirkova et al. “Sixteen years and counting: The current understanding of FGFR3 signaling in skeletal dysplasias”. In: *Human Mutation* 33.1 (2012), pp. 29–41. DOI: `https://doi.org/10.1002/humu.21636`.

[14] Cifuentes Laura Fontanals, Elisa Tonello, and Heike Siebert. “Control in Boolean Networks with Model Checking”. In: *Frontiers in Applied Mathematics and Statistics* 8 (Apr. 2022), p. 838546.

[15] Cifuentes Laura Fontanals et al. “Control Strategy Identification via Trap Spaces in Boolean Networks”. In: *CMSB*. Vol. 12314. LNCS. Springer. 2020, pp. 159–175.

[16] Luca Grieco et al. “Integrative modelling of the influence of MAPK network on cancer cell fate decision”. In: *PLOS Computational Biology* 9.10 (2013), e1003286.

[17] Matej Hajnal et al. “Toward Modelling and Analysis of Transient and Sustained Behaviour of Signalling Pathways”. In: *Hybrid Systems Biology*. Springer, 2016, pp. 57–66.

[18] Wilhelm Johannsen. “The genotype conception of heredity”. In: *The American Naturalist* 45.531 (1911), pp. 129–159.

[19] S.A. Kauffman. “Metabolic stability and epigenesis in randomly constructed genetic nets”. In: *Journal of Theoretical Biology* 22.3 (1969), pp. 437–467. ISSN: 0022-5193.

[20] John Kent. “Information gain and a general measure of correlation”. In: *Biometrika* 70.1 (Apr. 1983), pp. 163–173. ISSN: 0006-3444.

[21] Yunseong Kim et al. “Quantitative evaluation and reversion analysis of the attractor landscapes of an intracellular regulatory network for colorectal cancer”. In: *BMC systems biology* 11 (2017), pp. 1–11.

[22] Hannes Klarner et al. “Basins of attraction, commitment sets, and phenotypes of Boolean networks”. In: *IEEE/ACM TOCBB* 17.4 (2018), pp. 1115–1124.

[23] Santhana Kumar et al. “Discovery of a small molecule ligand of FRS2 that inhibits invasion and tumor growth”. In: *Cellular Oncology* 46.2 (2023), pp. 331–356.

[24] Alexander Lex et al. “UpSet: Visualization of Intersecting Sets”. In: *IEEE TACG* 20.12 (2014), pp. 1983–1992. DOI: 10.1109/TVCG.2014.2346248.

[25] Luis Fernando Méndez-López et al. “Gene regulatory network underlying the immortalization of epithelial cells”. In: *BMC systems biology* 11 (2017), pp. 1–15.

[26] Marija Milacic et al. “The Reactome Pathway Knowledgebase 2024”. In: *Nucleic Acids Research* 52.D1 (2023), pp. D672–D678. DOI: 10.1093/nar/gkad1025.

[27] Loïc Paulevé. “Marker and source-marker reprogramming of Most Permissive Boolean networks and ensembles with BoNesis”. In: *Peer Community Journal* 3 (2023).

[28] Dhruv Raina et al. “Intermittent ERK oscillations downstream of FGF in mouse embryonic stem cells”. In: 149.4 (2022), dev199710.

[29] Assieh Saadatpour et al. “Dynamical and Structural Analysis of a T Cell Survival Network Identifies Novel Candidate Therapeutic Targets for Large Granular Lymphocyte Leukemia”. In: *PLOS Computational Biology* 7.11 (2011), pp. 1–15.

[30] Anthony Stanislaus et al. "Knockdown of PLC-gamma-2 and calmodulin 1 genes sensitizes human cervical adenocarcinoma cells to doxorubicin and paclitaxel". In: *Cancer cell international* 12 (2012), pp. 1–8.

[31] Cui Su et al. "Scalable Control of Asynchronous Boolean Networks". In: *Computational Methods in Systems Biology*. Vol. 11773. Lecture Notes in Computer Science. Springer. 2019, pp. 364–367.

[32] Cui Su et al. "Sequential Temporary and Permanent Control of Boolean Networks". In: *Computational Methods in Systems Biology*. Vol. 12314. Lecture Notes in Computer Science. Springer. 2020, pp. 234–251.

[33] SM Minhaz Ud-Dean et al. "Optimal design of gene knockout experiments for gene regulatory network inference". In: *Bioinformatics* 32.6 (2016), pp. 875–883.

[34] José P Vaqué et al. "PLCG1 mutations in cutaneous T-cell lymphomas". In: *Blood* 123.13 (2014), pp. 2034–2043.

[35] Nguyen Xuan Vinh, Julien Epps, and James Bailey. "Information theoretic measures for clusterings comparison: is a correction for chance necessary?" In: *Proceedings of the 26th annual international conference on machine learning*. 2009, pp. 1073–1080.

[36] Peican Zhu et al. "Identification of potential drug targets in cancer signaling pathways using stochastic logical models". In: *Scientific reports* 6.1 (2016), p. 23078.
